# CtBP1/2 oligomerization promotes G9a-Mediated transcriptional repression

**DOI:** 10.1016/j.jbc.2025.111063

**Published:** 2025-12-17

**Authors:** Bin Zhang, Junheng Jiang, Wenxin Sun, Shengwei Hu, Peihan Chen, Linsheng Li, Meizhi Jiang, Junjie Chen, Jinzhang Zeng, Dachuan Cai, Qiang Luo, Wen Liu, Qixu Cai, Siming Chen

**Affiliations:** 1 School of Pharmaceutical Sciences, Fujian Provincial Key Laboratory of Innovative Drug Target Research, Xiamen University, Xiamen, China; 2 State Key Laboratory for Cellular Stress Biology, School of Life Sciences, Faculty of Medicine and Life Sciences, Xiamen University, Fujian, China; 3 School of Public Health, Xiamen University, Xiamen, China; 4 Department of Infectious Diseases, Institute for Viral Hepatitis, Key Laboratory of Molecular Biology for Infectious Diseases (Ministry of Education), The Second Affiliated Hospital, Chongqing Medical University, Chongqing, China; 5 Shenzhen Research Institute of Xiamen University, Shenzhen, Guangdong, China

**Keywords:** CtBP1/2, crystal structure, epigenetics, gene silencing, G9a, histone methyltransferase, protein oligomerization

## Abstract

Corepressors CtBP1 and CtBP2 (CtBP1/2) are evolutionarily conserved transcriptional regulators that repress gene expression by recruiting chromatin modifiers, yet the structural basis of this process remains elusive. Here, we identify a direct interaction between CtBP1/2 and the histone H3 lysine 9 (H3K9) methyltransferase G9a. Crystallographic and biochemical analyses reveal that a CtBP1/2 tetramer simultaneously engages two G9a molecules through a motif within the pre-SET domain of G9a, which is absent in its paralog GLP. This interaction enhances G9a catalytic activity in a manner strictly dependent on the oligomeric state of CtBP1/2. Disruption of CtBP2 tetramerization diminishes its association with G9a and abolishes enzymatic activation, underscoring the functional importance of CtBP1/2 oligomerization. In colorectal cancer (CRC) cells, CtBP2 and G9a co-occupy the PTEN promoter, where disruption of their interface reduces H3K9me2 deposition, derepresses PTEN expression, attenuates PI3K-AKT signaling, and impairs CRC cell proliferation. Together, these findings establish a structural framework for CtBP-mediated regulation of G9a activity and highlight the CtBP1/2-G9a complex as a potential therapeutic target in colorectal cancer.

In mammals, the C-terminal binding protein (CtBP) family consists of two closely related paralogues, CtBP1 and CtBP2, which function as redundant transcriptional co-regulators (1). These evolutionarily conserved proteins regulate gene expression by shaping chromatin architecture through facilitating histone modifications, nucleosome positioning, and modulating transcription factor activity (2, 3, 4, 5). Unlike many classical transcriptional regulators, CtBP1/2 lack intrinsic DNA-binding domains and are recruited to specific genomic loci *via* interactions with sequence-specific transcription factors (6). This reliance on protein–protein interactions indicates that the transcriptional output of CtBP1/2 is determined primarily by their repertoire of binding partners. Therefore, the systematic identification and characterization of CtBP1/2-associated proteins are essential for elucidating the molecular mechanisms by which CtBP1/2 exert their regulatory functions.

Emerging evidence indicates that CtBP1/2 can assemble into large transcriptional complexes with both repressive and activating functions (6, 7, 8). Co-immunoprecipitation and proximity-labeling studies have revealed CtBP1/2 associations with several key chromatin modifiers, including the H3K9 methyltransferases G9a/GLP (9), the H3K4/9 demethylase LSD1 (10), histone deacetylases (HDACs) (11, 12, 13), and Polycomb-associated factors such as CDYL (14, 15). However, most of these interactions have been inferred from bulk biochemical purifications or chromatin co-occupancy analyses, leaving two critical questions unresolved: (i) do CtBP1/2 engage these epigenetic writers and erasers through direct physical interactions or *via* bridging adaptors? and (ii) how does CtBP1/2 binding influence their enzymatic activities? Addressing these questions will require systematic domain-mapping, high-resolution structural studies, and enzymatic assays to move beyond correlative co-recruitment and delineate the molecular grammar linking CtBP1/2 to chromatin modification.

CtBP1/2 exist in a dynamic equilibrium among monomeric, dimeric, and higher-order oligomeric states (2, 16, 17, 18), with transcriptional co-repressor activity requiring at least dimerization or oligomerization (8, 14, 19). NAD(H)-binding promotes CtBP2 oligomerization, whereas acyl-CoA binding disrupts oligomer formation and stabilizes the monomeric state (20, 21, 22). This metabolite-dependent conformational regulation modulates CtBP interactions with distinct transcription factors and thereby fine-tunes transcriptional output (8, 21, 23). For example, acyl-CoA–induced monomeric CtBP2 preferentially associates with PPARα (22), whereas NADH-driven oligomerization enhances binding to FOXO1 (21). These findings highlight that specific oligomeric states are coupled to distinct transcriptional outcomes (8). Nevertheless, while the role of CtBP oligomerization in transcriptional regulation has become increasingly appreciated, how different oligomeric states influence the activities of chromatin modifiers to regulate gene expression remains largely unknown.

G9a (EHMT2) is a SET-domain-containing histone methyltransferase that catalyzes mono- and dimethylation of histone H3 at lysine 9 (H3K9me1/2) in euchromatic regions, thereby promoting transcriptional repression (24, 25). In mammalian cells, G9a functions either as a homodimer or as a heterodimer with GLP (EHMT1), another SET-domain protein, and such dimeric assemblies are essential for its biological activity *in vivo* (9, 26, 27, 28). G9a and GLP serve as core components of multiple transcriptional repression complexes, including those associated with E2F6, CtBP1/2, and CDP/cut, and they also reside within large (∼1 MDa) assemblies in embryonic stem cells (29, 30, 31, 32). Previous studies have suggested that G9a activity may be directly regulated by CtBP1/2 (7, 15). However, other reports indicate that CtBP1/2 interact with G9a indirectly through the zinc finger protein Wiz (29). Thus, it remains unclear whether CtBP1/2 can directly associate with the G9a/GLP complex and, if so, whether such interactions modulate G9a′s enzymatic activity in the regulation of gene expression.

Here, we combine X-ray crystallography, biochemical assays, and cellular functional studies to establish how CtBP1/2 directly govern G9a activity. We report the 1.85 Å structure of a tetrameric CtBP1/2 core that simultaneously engages two G9a molecules through a previously uncharacterized motif within the G9a pre-SET domain. This motif, absent in the GLP pre-SET domain, underlies the structural basis of a selective and stoichiometric corepressor-enzyme complex. Biochemical analyses reveal that CtBP1/2 allosterically enhance G9a catalytic activity in an oligomerization-dependent manner, whereas oligomerization-deficient mutants fail to promote methylation. In colorectal cancer cells, disrupting the CtBP2-G9a interface reduces H3K9me2 deposition at target promoters such as PTEN, leading to transcriptional derepression, attenuation of oncogenic signaling, and impaired tumor cell proliferation. Collectively, our findings demonstrate that higher-order assembly of CtBP1/2 couples transcriptional repression to H3K9 methylation, providing a mechanistic framework for CtBP1/2-mediated epigenetic regulation and highlighting the therapeutic potential of targeting the CtBP-G9a complex in colorectal cancer.

## Results

### The G9a Pre-SET domain mediates direct interaction with CtBP1/2

**Figure 1:**
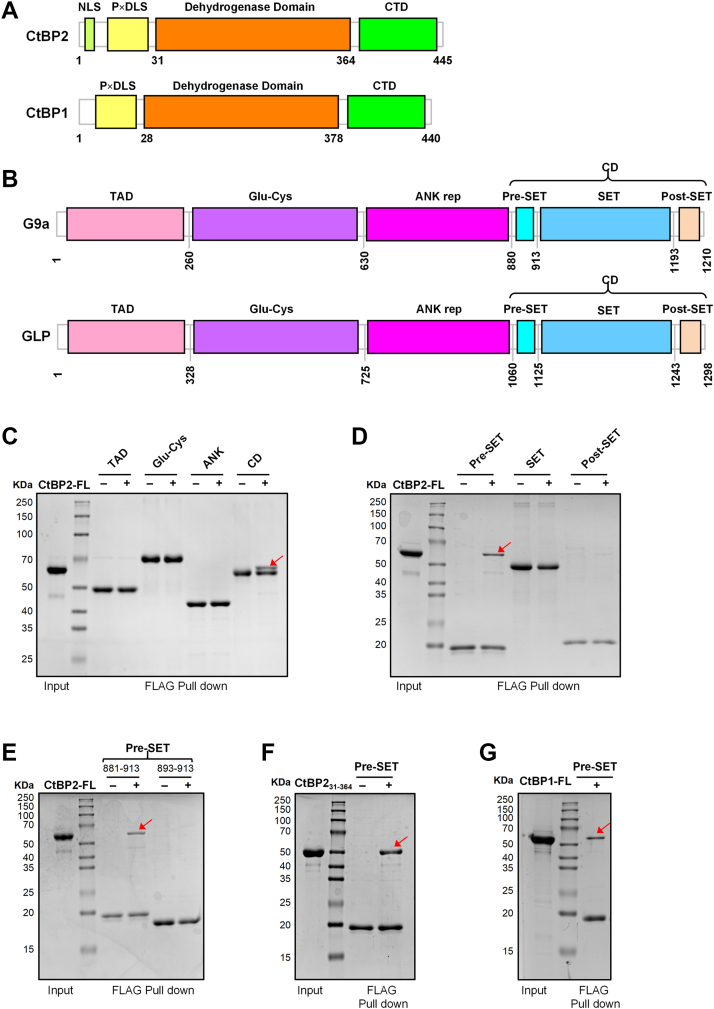
**G9a interacts with CtBP2/1 through its Pre-SET domain.***A*, schematic representation of human CtBP2 and CtBP1 domain organization. *B*, domain architecture of human G9a and GLP. Numbers indicate amino acid positions. *C*, mapping of the CtBP2-binding region on G9a using FLAG pull-down assays. Equal amounts of the indicated SUMO-G9a-FLAG fragments (TAD, 1–260; Glu-Cys, 261–630; ANK, 631–880; CD, 881–1210) were immobilized on anti-FLAG resin. Only the CD fragment (aa 881–1210) retained CtBP2 under identical experimental conditions. *D*, Pull-down assays using the Pre-SET (881–913), SET (913–1193), and post-SET (1193–1210) fragments. CtBP2 binding was detected exclusively with the Pre-SET fragment. *E*, determination of the minimal Pre-SET motif required for CtBP2 interaction. SUMO-G9a (881–913)-FLAG and the shorter SUMO-G9a (893–913)-FLAG were tested. Deletion of residues 881 to 892 abolished binding, indicating that the N-terminal portion of Pre-SET is essential. *F*, direct interaction between minimal fragments of G9a and CtBP2. SUMO-G9a (881–913)-FLAG efficiently pulled down the CtBP2 dehydrogenase domain (aa 31–364), confirming a direct interaction. *G*, conserved recognition of CtBP1. The G9a Pre-SET fragment (881–913) also captured full-length SUMO-CtBP1, demonstrating that both CtBP paralogs share the same docking site.

G9a is a SET domain-containing histone methyltransferase organized into four distinct modules: an N-terminal transcriptional activation domain (TAD, residues 1–260), a central glutamate–cysteine–rich region (Glu-Cys, residues 261–630), an ankyrin repeat domain (ANK, residues 631–880), and a C-terminal catalytic domain (CD, residues 881–1210) (Fig. 1*B*) (33). To determine whether G9a directly interacts with CtBP1/2, each of these four fragments was expressed in *Escherichia coli* as an N-terminal SUMO-tagged, C-terminal FLAG-tagged fusion protein and purified to homogeneity. Full-length CtBP2, fused to an N-terminal SUMO tag, was similarly expressed and purified (Fig. S1*A*). *In vitro* FLAG pull-down assays demonstrated that only the CD-containing fragment of G9a bound CtBP2, whereas the TAD, Glu-Cys, and ANK domains showed no detectable association (Fig. 1,*C*).

The C-terminal CD region of G9a can be further subdivided into three domains: Pre-SET (residues 881–913), SET (residues 913–1193), and Post-SET (residues 1193–1210) (Fig. 1*B*) (34, 35, 36). The SET domain harbors the catalytic methyltransferase activity of G9a (25, 37). To pinpoint the domain responsible for CtBP1/2 interaction, we generated a series of G9a truncations and performed pull-down assays. Only the Pre-SET fragment retained binding to CtBP2, whereas neither the SET nor the Post-SET domains showed detectable association (Fig. 1*D*). Further mapping revealed that the Pre-SET domain (residues 881–913) was sufficient for CtBP2 binding, but a shorter fragment (residues 893–913) failed to interact, indicating that residues 881 to 893 are essential for mediating the G9a-CtBP2 association (Fig. 1*E*).

CtBP2 is composed of 445 amino acids, with both its N-terminal 30 residues and C-terminal 81 residues predicted to be intrinsically disordered (Fig. 1*A*) (38, 39, 40). To facilitate crystallographic studies, we generated an N- and C-terminally truncated CtBP2 construct (residues 31–364). FLAG pull-down assays confirmed that the G9a Pre-SET fragment bound directly to CtBP2(31–364) (Fig. 1*F*). Given the high degree of sequence conservation between CtBP1 and CtBP2 (Fig. S2), we also expressed and purified full-length CtBP1 (Fig. S1*B*) and verified by pull-down assays that the G9a Pre-SET fragment directly associates with CtBP1 (Fig. 1*G*).

GLP (EHMT2), a close paralog of G9a (EHMT1), exhibits extensive structural and functional conservation with G9a (25, 41, 42). Sequence alignment revealed that the Pre-SET domain of GLP lacks the DTAWDLTPE motif, which is essential for G9a–CtBP2 interaction (Fig. S3*B*). Interestingly, the Glu-Cys region of GLP harbors a potential CtBP-binding DLS motif that is absent from the corresponding region of G9a (Fig. S3*A*). Based on these observations, we hypothesized that GLP might recruit CtBP2 *via* its DLS-containing Glu-Cys domain rather than through the Pre-SET element used by G9a. Consistent with this prediction, FLAG pull-down assays showed that the Glu-Cys fragment of GLP robustly associated with CtBP2 (Fig. S3*D*), whereas the Pre-SET fragment exhibited no detectable binding (Fig. S3*C*). Collectively, these results indicate that, despite their homology, G9a and GLP engage CtBP2 through distinct surfaces: G9a relies on its Pre-SET motif, whereas GLP depends on the DLS motif within its Glu-Cys domain.

### Crystal structure of CtBP2 in complex with the G9a Pre-SET domain

**Figure 2:**
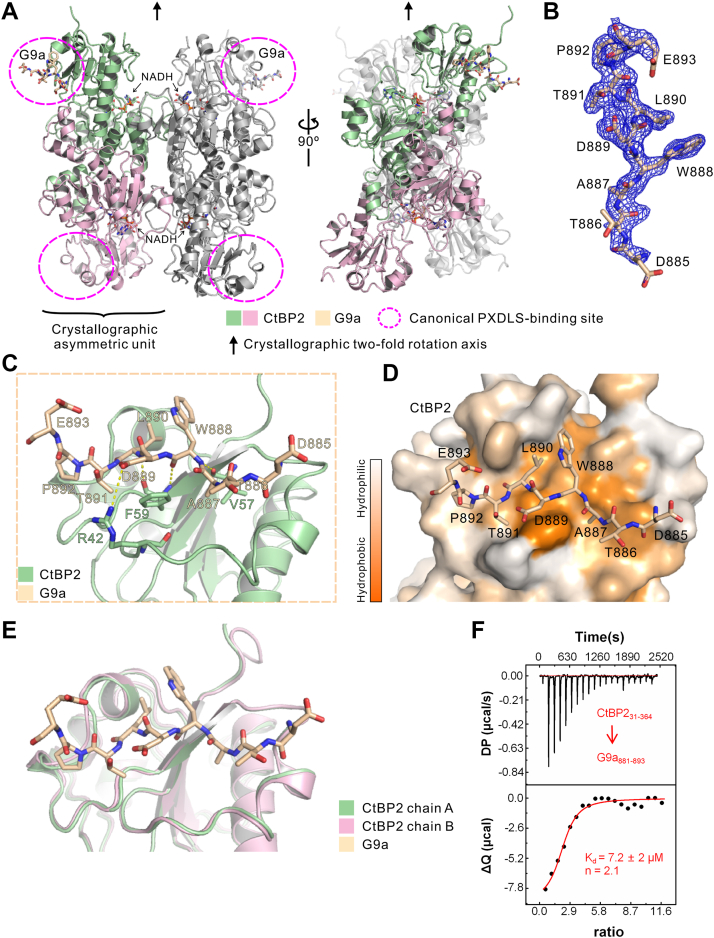
**Crystal structure of CtBP2 in complex with the G9a peptide.***A*, cartoon representation of the CtBP2-G9a complex. Crystallographic symmetry-related molecules are shown in gray. *B*, final 2mFo-DFc electron density map contoured at 1.0 σ, with the G9a peptide shown in stick representation. *C*, detailed view of the interaction interface between CtBP2 and the G9a peptide. *D*, surface representation of CtBP2 with the G9a peptide shown as sticks, highlighting hydrophobic interactions that stabilize the complex. *E*, superposition of chain A and chain B from the CtBP2-G9a structure, showing their high structural similarity. *F*, isothermal titration calorimetry (ITC) analysis of CtBP2 binding to the G9a peptide.

To elucidate the structural basis of the G9a-CtBP2 interaction, our previous alanine-deletion mapping demonstrated that the Pre-SET domain of G9a (residues 881–893) is essential for CtBP2 binding (Fig. 1*E*). Guided by this result, we chemically synthesized a G9a peptide encompassing residues 881 to 893 and expressed and purified human CtBP2 (residues 31–364) from *E. coli* (Fig. S4, *A* and *B*). Co-crystallization of CtBP2 with the G9a peptide yielded well-diffracting crystals, and the structure was solved at 1.85 Å resolution (Fig. 2 and Table S3). Surprisingly, each asymmetric unit contains a 2:1 stoichiometric complex of CtBP2 and the G9a peptide (Fig. 2*A*). Electron densities are well-defined for most residues of both molecules in the complex (Fig. 2*B*). Consistent with previous reports (16, 17, 43), CtBP2 assembles into a tetramer in the crystal, with the G9a peptide bound to two of the four canonical PXDLS-binding sites (Fig. 2*A*). The G9a peptide adopts a canonical CtBP2-binding mode *via* the PXDLS motif. Specifically, the CtBP2-G9a interaction is mediated by extensive hydrogen bonding between the main chains of both proteins, including F59_CtBP2_-L890_G9a_, F59_CtBP2_-W888_G9a,_ and V57_CtBP2_-W888_G9a_ (Fig. 2*C*). Electrostatic interactions between the side chains of R42_CtBP2_ and D889_G9a_ further stabilize the complex (Fig. 2*A*). In addition, hydrophobic residues W888 and L890 of G9a interact with a complementary hydrophobic surface on CtBP2, strengthening the binding (Fig. 2*D*).

Interestingly, in the asymmetric unit, only chain A directly engages the G9a peptide, while chain B does not, resulting in a tetrameric CtBP2 bound to two G9a peptides (Fig. 2*A*). Despite the absence of G9a peptide density in chain B, its conformation closely resembles that of chain A, with a root-mean-square deviation of 0.45 Å between the Cα atoms of the two chains (Fig. 2*E*). Consistent with the structural observations, isothermal titration calorimetry (ITC) confirmed a 2:1 binding stoichiometry between CtBP2 and the G9a peptide (Fig. 2*F*). This stoichiometry matches that observed in the crystal structure of the CtBP2-G9a complex.

**Figure 3:**
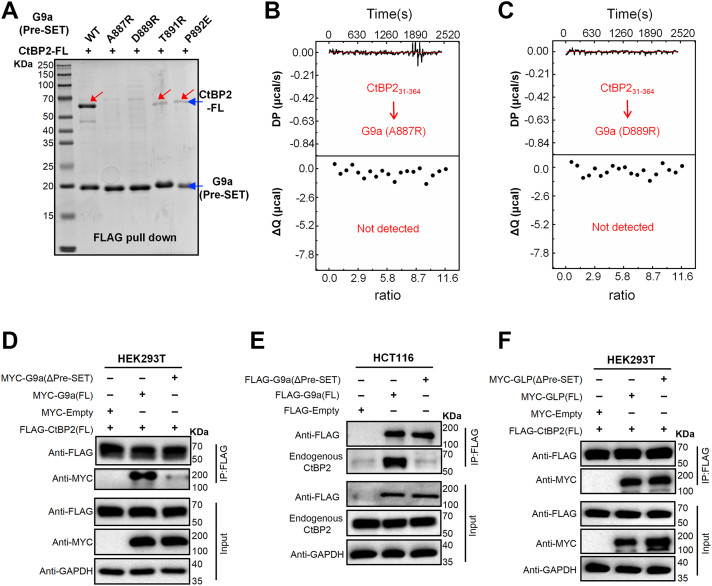
**Mutations at the interface impair CtBP2–G9a association *in vitro* and in cells.***A*, *in vitro* FLAG pull-down. Equal amounts of SUMO-G9a^881–913^-FLAG (wild type or mutants A887R, D889R, T891R, P892E) were immobilized on anti-FLAG resin and incubated with equal amounts of SUMO-CtBP2-FL. A887R and D889R abolished binding, while T891R and P892E reduced binding to a lesser extent (*upper blot*). Ponceau staining confirmed equal bait loading (*lower panel*). *B* and *C*, ITC analysis. CtBP2^31–364^ (2 mM) was titrated into G9a^881–893^ peptides (25 μM) carrying A887R (*B*) or D889 R (*C*). No heat release was detected, indicating undetectable affinity. *D*, Co-IP in HEK293T cells. Cells co-transfected with CtBP2-FLAG and MYC-tagged G9a-FL, G9a-FL (ΔPre-SET, Δ885–893), or empty vector. Full-length G9a co-precipitated CtBP2, whereas the ΔPre-SET(Δ885–893) G9a mutants did not. *E*, endogenous interaction in HCT116 cells. FLAG-IP from stable cells expressing 3 × FLAG-G9a^WT^ or 3 × FLAG-G9a (ΔPre-SET, Δ885–893) showed that only wild-type G9a retrieved endogenous CtBP2. *F*, Co-IP in HEK293 T cells. CtBP2-FLAG was co-expressed with MYC-tagged GLP-FL, GLP-FL (ΔPre-SET, Δ885–893), or empty vector. Both full-length and ΔPre-SET(Δ885–893) GLP co-precipitated CtBP2.

To validate the CtBP2-G9a complex structure, we introduced point mutations into the G9a Pre-SET domain and evaluated their effects on CtBP2 binding. Both FLAG pull-down assays and ITC experiments revealed that the A887R and D889R mutations completely abolished the interaction, likely due to steric hindrance between the mutated residues and CtBP2 (Fig. 3, *A*–*C*). The T891R and P892E mutants exhibited substantially reduced binding (Fig. 3*A*). We then examined the functional relevance of this motif in the context of full-length G9a in cells. HEK293T cells were co-transfected with full-length FLAG-CtBP2 and either Myc-G9a-FL or Myc-G9a^Δ885–893^ (lacking the DTAWDLTPE motif). Co-immunoprecipitation (Co-IP) assays showed that wild-type G9a robustly associates with CtBP2, whereas the deletion mutant completely lost this interaction (Fig. 3*D*). To further corroborate these findings under more physiological conditions, we generated HCT116 cell lines stably expressing 3 × FLAG-tagged wild-type G9a or G9a^Δ885–893^. Endogenous Co-IP experiments demonstrated that only wild-type G9a co-precipitated with endogenous CtBP1/2, whereas the deletion mutant failed to do so (Fig. 3*E*). Collectively, these biochemical and cellular results indicate that the DTAWDLTPE motif (G9a residues 885–893) is both necessary and sufficient for G9a to engage CtBP1/2.

To investigate whether the distinct CtBP-binding modes of G9a and GLP observed *in vitro* are maintained in cells, we conducted co-immunoprecipitation assays in HEK293 T cells. Consistent with our biochemical mapping, FLAG-CtBP2 interacted robustly with full-length Myc-GLP, and deletion of the entire Pre-SET domain (GLP^ΔPre-SET^) had no impact on this association (Fig. 3*F*). In contrast, the same deletion completely abolished binding to G9a (Fig. 3, *D* and *E*), indicating that the two paralogs employ different structural elements to recruit CtBP1/2 in cells, with G9a relying on its Pre-SET domain and GLP on the Glu-Cys region containing the DLS motif. Together, these findings reveal that, despite overlapping catalytic activities, G9a and GLP have evolved distinct molecular strategies to engage the CtBP co-repressor complex.

### CtBP2 oligomerization enhances G9a methyltransferase activity

**Figure 4:**
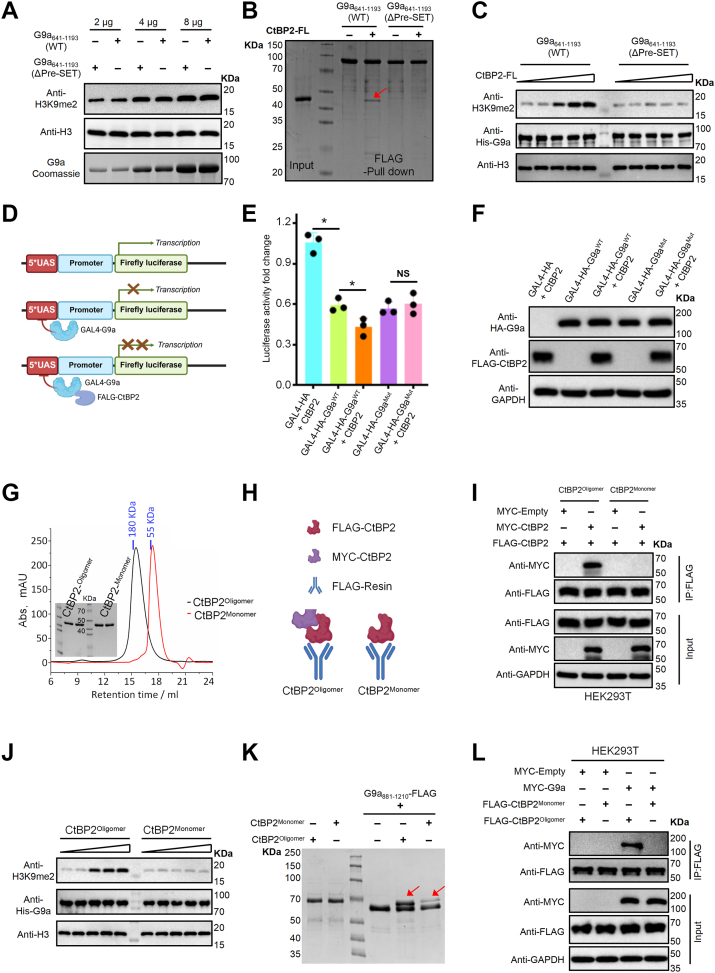
**Oligomerization of CtBP2 potentiates G9a methyltransferase activity.***A*, *in vitro* methyltransferase assay with increasing amounts (2, 4, 8 μg) of His6-MBP-G9a^641-1193^ (WT) or His6-MBP-G9a^ΔPre-SET^ (Δ885–893). Coomassie staining confirms equal loading; immunoblots show H3K9me2 and total H3. Both fragments exhibit comparable basal activity. *B*, MBP pull-down assay with full-length CtBP2. Only WT G9a retrieves CtBP2, identifying residues 885 to 893 as the docking site. *C*, CtBP2-FL stimulates G9a activity in a dose-dependent manner. Reactions contained 8 μg G9a (WT or ΔPre-SET), 0.25 μg H3, 32 μM SAM, and 0 to 4 μg CtBP2-FL at 30 °C for 4 h H3K9me2 levels increase only with WT G9a. *D*, luciferase reporter schematic: five GAL4-UAS repeats drive Firefly luciferase under the minimal UHRF1 promoter; GAL4-HA-G9a (WT or Δ885–893) recruitment reports G9a-mediated repression. *E*, relative luciferase activity in HEK293T cells expressing combinations of GAL4-HA-G9a and CtBP2-FL-FLAG, normalized to vector controls. Bar heights represent the mean values from three independent luciferase assays, and error bars denote the standard deviation (SD). Statistical significance was determined using unpaired t-tests or one-way ANOVA, as appropriate. n.s., not significant; *p* < 0.05 (∗); *p* < 0.01 (∗∗). (n = 3 biological replicates). *F*, Western blot confirming comparable expression of G9a and CtBP2 constructs used in (*D* and *E*); GAPDH as loading control. *G*, size-exclusion chromatography of CtBP2 on a Superose six column. Wild-type CtBP2 elutes as a broad peak corresponding to oligomers (∼180 kDa), whereas the quadruple mutant (R147A/R148A/R169A/R177A) elutes at ∼55 kDa, consistent with a monomeric state. Fractions were analyzed by SDS-PAGE. *H* and *I*, two-tag co-immunoprecipitation. FLAG-CtBP2 WT pulls down MYC-CtBP2 WT, whereas monomeric FLAG-CtBP2 fails to retrieve MYC-CtBP2 monomer, indicating self-association requires the oligomeric form. *J*, G9a recruitment assay: only oligomeric CtBP2 efficiently captures MYC-G9a. *K*, *in vitro* FLAG pull-down of SUMO-G9a^881–1210^-FLAG with WT or monomeric CtBP2. Robust binding observed for WT, minimal for monomer. *L*, oligomerization-dependent stimulation of G9a methyltransferase activity. H3K9me2 levels increase only with oligomeric CtBP2, indicating that higher-order CtBP2 assembly is required for maximal activity.

G9a is a histone methyltransferase that catalyzes mono- and dimethylation of histone H3 lysine 9 (H3K9me1/2), thereby contributing to transcriptional repression (24, 25). Our structural and biochemical analyses revealed that G9a directly interacts with CtBP1/2 through its Pre-SET domain. These observations raised two key questions: (i) does CtBP1/2 modulate G9a enzymatic activity, and (ii) is this regulation dependent on the Pre-SET domain? To address these questions, we first assessed whether the Pre-SET domain itself contributes to G9a catalytic activity. To obtain enzymatically active G9a in *E. coli*, we generated two constructs: G9a^641-1193^(WT), comprising the ankyrin (ANK), Pre-SET, and SET domains, and G9a^641-1193^(ΔPre-SET), a Pre-SET-deleted version of the same fragment. Both proteins were efficiently expressed in *E. coli* and purified to homogeneity. In *in vitro* methyltransferase assays performed under identical protein concentrations, G9a^641-1193^(WT) and G9a^641-1193^(ΔPre-SET) exhibited comparable catalytic activities (Fig. 4*A*). These results demonstrate that the Pre-SET domain is dispensable for the intrinsic enzymatic activity of G9a.

To determine whether the CtBP2-G9a interaction translates into enzymatic stimulation, we performed *in vitro* methyltransferase assays using purified recombinant CtBP2 together with either G9a^641-1193^(WT) or G9a^641-1193^(ΔPre-SET). Stepwise addition of CtBP2 enhanced the catalytic activity of WT G9a in a concentration-dependent manner, whereas the ΔPre-SET mutant remained unresponsive under the same conditions (Fig. 4, *B* and *C*). These results demonstrate that the Pre-SET domain, which mediates CtBP2 binding, is required for activation of G9a. We next examined whether this regulatory mechanism operates in a cellular context. A Gal4-luciferase reporter with five Gal4-binding sites upstream of the UHRF1 promoter, previously used to monitor G9a-mediated transcriptional repression, was co-transfected with Gal4-DBD-G9a (WT or ΔPre-SET) in the presence or absence of CtBP2 (44). CtBP2 significantly increased the repressive activity of G9a(WT) but failed to enhance silencing by G9a(ΔPre-SET) (Fig. 4, *D* and *E*). Importantly, in the absence of CtBP2, both G9a variants repressed reporter activity to a comparable degree (Fig. 4*E*), indicating that the observed differences are attributable to CtBP2 regulation rather than intrinsic catalytic capacity. Collectively, these findings establish that direct engagement of the G9a Pre-SET domain by CtBP2 is essential for stimulation of methyltransferase activity *in vitro* and for potentiation of transcriptional repression in cells.

CtBP1/2 exist in a dynamic equilibrium between monomeric, dimeric, and higher-order oligomeric states in response to cellular metabolic conditions (2, 20, 21, 22). Previous studies have established that oligomerization is indispensable for their transcriptional repressor functions, although the precise molecular mechanism remains unclear (8, 14, 19). In our crystal structure of the CtBP2-G9a complex, CtBP1/2 assemble as tetramers that simultaneously engage two G9a molecules (Fig. 2*A*), suggesting that oligomerization may underlie their regulatory effect on G9a activity. To test this hypothesis, we generated a monomeric CtBP2 variant (hereafter referred to as CtBP2^Monomer^) by substituting four conserved residues (R147A, R148A, R169A, and R177A) previously shown to disrupt CtBP1 self-association (14, 45, 46). Wild-type CtBP2 is hereafter referred to as CtBP2^Oligomer^. Analytical size-exclusion chromatography was performed to assess the oligomeric states of the two variants. CtBP2^Monomer^ eluted at a position corresponding to a ∼50 kDa species, consistent with a monomeric state, whereas CtBP2^Oligomer^ eluted at ∼180 kDa, consistent with a tetrameric assembly (Fig. 4*G*). Supporting this, two-tag co-immunoprecipitation assays in HEK293T cells confirmed that, compared to wild-type CtBP2, the four-residue mutation effectively abolished self-association, yielding a monomeric form (Fig. 4, *H* and *I*).

To evaluate whether CtBP2 oligomerization is required for stimulation of G9a methyltransferase activity, we first performed *in vitro* assays, which demonstrated that oligomeric CtBP2 (CtBP2^Oligomer^), but not the monomeric mutant (CtBP2^Monomer^), enhanced G9a-catalyzed H3K9me1/2 deposition (Fig. 4*J*). Consistent with this functional difference, pull-down assays and co-immunoprecipitation experiments revealed that CtBP2^Monomer^ exhibits markedly reduced binding to G9a compared with CtBP2^Oligomer^ (Fig. 4, *K* and *L*), indicating that oligomerization strengthens CtBP2-G9a interactions.

To determine whether this oligomerization-dependent regulation also operates in living cells, we next conducted Gal4-luciferase reporter assays under three conditions: G9a(WT) alone, G9a(WT) with CtBP2^Oligomer^, and G9a(WT) with CtBP2^Monomer^. In line with our biochemical findings (Fig. 4, *J*–*L*), CtBP2^Oligomer^ markedly enhanced G9a(WT)-mediated transcriptional repression, whereas CtBP2^Monomer^ failed to exert any detectable effect (Fig. S4, *C* and *D*). These combined results demonstrate that CtBP2 oligomerization is indispensable for potentiating G9a-dependent transcriptional repression in cells, reinforcing the model that oligomeric CtBP2 complexes promote G9a recruitment and activation to enforce transcriptional silencing.

### Disruption of the CtBP2-G9a interface restrains CRC cell proliferation

G9a is frequently amplified or transcriptionally upregulated in colorectal cancer (CRC), where elevated expression correlates with advanced TNM stage, metastasis, and poor survival (47, 48, 49). Mechanistically, G9a-mediated deposition of H3K9me1/2 represses tumor-suppressor genes such as CDKN1A/p21, E-cadherin, and PTEN, thereby promoting cell proliferation, epithelial-to-mesenchymal transition (EMT), and chemoresistance (30, 50, 51, 52). CtBP2 is likewise frequently overexpressed in CRC and enriched in tumor-initiating cell (TIC) populations (11, 53). As a metabolic sensor, CtBP2 responds to NAD^+^/NADH fluctuations by oligomerizing into transcriptional repressor complexes that coordinate oncogenic signaling pathways such as Wnt/β-catenin, Notch, and PI3K-AKT. Genetic depletion of CtBP2 has been shown to reduce tumourigenicity in patient-derived xenografts (54, 55, 56). Our crystallographic and biochemical analyses now reveal that tetrameric CtBP2 directly engages the pre-SET domain of G9a to stimulate its methyltransferase activity, thereby providing a molecular link between metabolic sensing and epigenetic silencing. Based on these findings, we hypothesized that the CtBP2-G9a interaction cooperates to drive CRC progression and that pharmacological or genetic disruption of this interface may restore tumor-suppressor gene expression and attenuate malignant proliferation.

**Figure 5:**
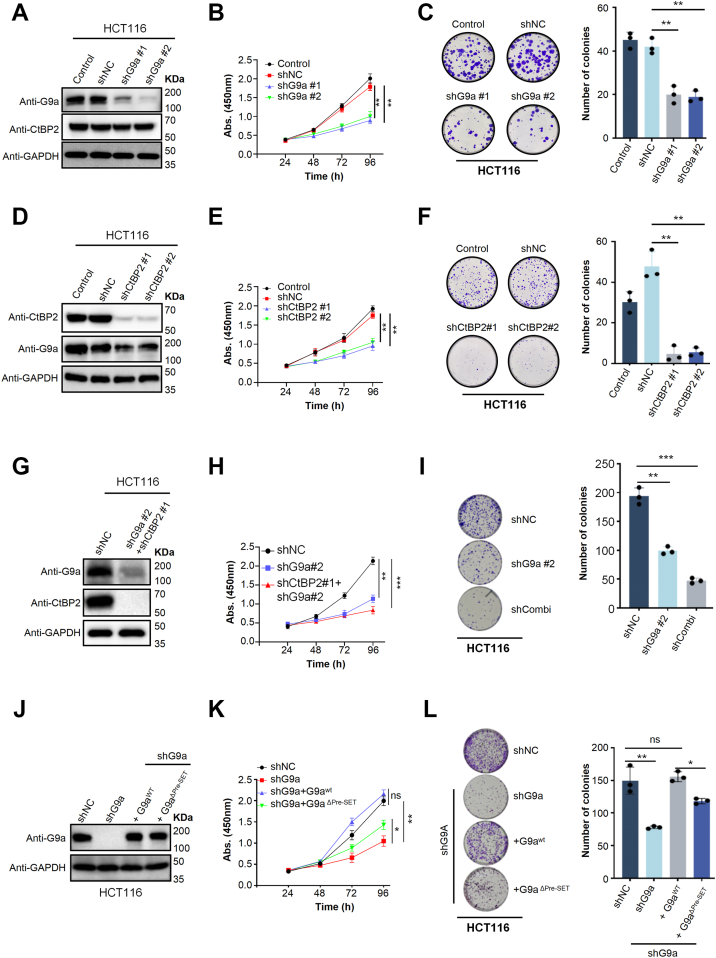
**Disruption of the G9a-CtBP2 interaction suppresses colorectal cancer cell growth.***A*, Western blot confirming G9a knockdown in HCT116 cells. Cells were infected with two independent shG9a hairpins (#1, #2) or non-targeting shNC control. GAPDH serves as loading control. *B* and *C*, colony formation and proliferation assays in HCT116 cells. Compared with untreated or shNC cells, G9a depletion (shG9a#1 or shG9a#2) significantly reduces cell growth (*p* < 0.01, one-way ANOVA). Data were analyzed using unpaired t-tests or one-way ANOVA, as appropriate. n.s., not significant; *p* < 0.05 (∗); *p* < 0.01 (∗∗). Data are presented as mean ± SD from three independent experiments. *D*, Western blot confirming CtBP2 knockdown using two independent shCtBP2 hairpins (#1, #2); GAPDH serves as loading control. *E* and *F*, CtBP2 silencing phenocopies G9a loss, with significant reductions in cell proliferation (*E*) and colony formation (*F*) (*p* < 0.01, one-way ANOVA). Statistical analyses were performed using unpaired t-tests or one-way ANOVA, as appropriate. n.s., not significant; *p* < 0.05 (∗); *p* < 0.01 (∗∗). Data are presented as mean ± SD from three independent experiments. *G*, double knockdown of CtBP2 and G9a. Co-expression of shCtBP2#1 and shG9a#2 results in near-complete loss of both proteins, normalized to GAPDH. *H* and *I*, combined depletion produces additive growth inhibition, suggesting that CtBP2 and G9a cooperate to support colorectal cancer cell growth. Statistical analyses were performed using unpaired t-tests or one-way ANOVA, as appropriate. n.s., not significant; *p* < 0.05 (∗); *p* < 0.01 (∗∗); *p* < 0.001 (∗∗∗). Data are presented as mean ± SD from three independent experiments. *J*, rescue experiment. shG9a#2 cells were reconstituted with RNAi-resistant constructs: G9a^WT^-FLAG or G9a^ΔPre-SET^-FLAG (Δ885–893). Expression levels of reconstituted proteins approach endogenous G9a levels in shNC cells (GAPDH control). *K* and *L*, only wild-type G9a restores proliferation and colony formation. HCT116 cells expressing G9a^WT^, but not the G9a^ΔPre-SET^ mutant, rescue cell growth. Statistical analysis was performed using one-way ANOVA. Statistical analyses were performed using unpaired t-tests or one-way ANOVA, as appropriate. n.s., not significant; *p* < 0.05 (∗); *p* < 0.01 (∗∗). Data are presented as mean ± SD from three independent experiments.

To address this hypothesis, we first investigated the functional contribution of G9a and CtBP2 to CRC cell growth. Stable knockdown of G9a using two independent shRNAs (Fig. 5*A*) resulted in a pronounced reduction of HCT116 cell proliferation, as determined by CCK-8 viability assays and clonogenic growth (Fig. 5, *B* and *C*). Consistently, depletion of CtBP2 similarly suppressed cell proliferation (Fig. 5, *D*–*F*). Notably, combined silencing of G9a and CtBP2 produced a significantly greater inhibition of both cell viability and colony-forming capacity than either single knockdown alone (Fig. 5, *H* and *I*). These results demonstrate that G9a and CtBP2 are each required for optimal CRC cell growth and further suggest that they act in a cooperative manner to sustain malignant proliferation.

Structural analyses revealed that G9a engages CtBP2 through the DTAWDLTPE motif within its pre-SET domain, and deletion of this motif (G9a^ΔPre-SET^) abolished CtBP2 binding both *in vitro* and in cells. To directly test whether the pro-proliferative activity of G9a and CtBP2 requires their physical association, we reintroduced shRNA-resistant full-length wild-type G9a (G9a^WT^) or the binding-deficient G9a^ΔPre-SET^ mutant into G9a-depleted HCT116 cells. As expected, expression of G9a^WT^ fully rescued cell proliferation, restoring short-term viability as well as long-term clonogenic growth (Fig. 5, *K* and *L*). By contrast, G9a^ΔPre-SET^ supported only partial growth recovery, despite comparable expression levels (Fig. 5*J*). These results establish that the direct CtBP2-G9a interaction is indispensable for maximal proliferative output in CRC cells, and that disruption of this interface markedly compromises tumor cell growth.

Given the essential role of CtBP2 oligomerization in maintaining its stable association with G9a, we next asked whether CRC cell proliferation similarly relies on the oligomeric state of CtBP2. To test this, we performed rescue experiments in CtBP2-knockdown cells and found that reintroduction of wild-type CtBP2 (CtBP2^Oligomer^) fully restored proliferation, whereas the oligomerization-defective mutant (CtBP2^Monomer^) failed to rescue the growth defect, phenocopying the shCtBP2 control (Fig. S5, *B* and *C*). These results establish that CtBP2 oligomerization is strictly required for the G9a-dependent transcriptional program that drives CRC cell proliferation. Together with our biochemical and structural data, these findings demonstrate that CtBP2-mediated stimulation of G9a constitutes a critical epigenetic mechanism driving transcriptional silencing in colorectal cancer.

### Disruption of the CtBP2-G9a axis rewires PTEN-AKT signaling in CRC

**Figure 6:**
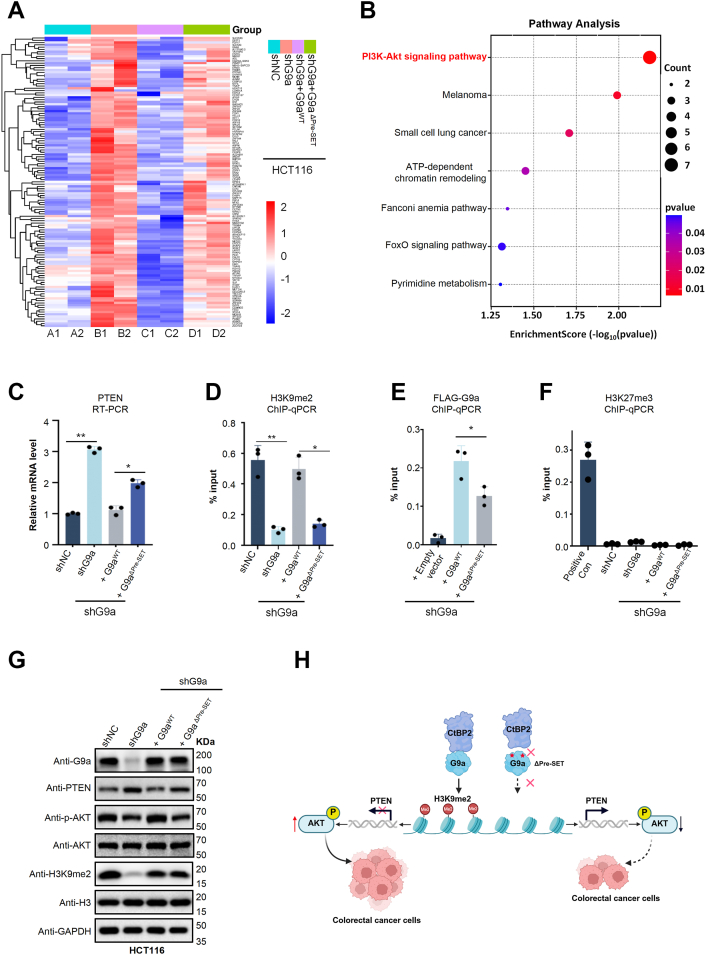
**Disruption of the G9a-CtBP2 interaction attenuates PI3K-AKT signaling.***A*, heat map of genes co-regulated by G9a and CtBP2 in HCT116 cells. *B*, KEGG pathway enrichment of the genes in (*A*) (SRplot). The PI3K-AKT pathway is most significantly enriched (adjusted *p* < 0.05). *C*, qPCR quantification of PTEN mRNA in HCT116 cells expressing control shRNA (shNC), shG9a#2, or shG9a#2 rescued with either wild-type G9a (G9a^WT^) or the CtBP2-binding mutant (G9a^ΔPre-SET^, Δ885–893). Data are mean ± s.d. (n = 3 biological replicates); ∗∗*p* < 0.01, n.s. = not significant (one-way ANOVA). *D–F*, ChIP–qPCR assays at the PTEN promoter. *D*, H3K9me2 occupancy in the cell lines described in (*C*). *E*, recruitment of G9a^WT^*versus* G9a^ΔPre-SET^. *F*, H3K27me3 occupancy in the same cell lines. The CDKN1A promoter was included as a positive control for H3K27me3 enrichment. Values are presented as % input. Bar heights represent mean values from three independent experiments, and error bars indicate the standard deviation (SD). Statistical analyses were performed using unpaired t-tests or one-way ANOVA, as appropriate. n.s., not significant; *p* < 0.05 (∗); *p* < 0.01 (∗∗). (n = 3 biological replicates). *G*, Western blot analysis of whole-cell lysates probed for PTEN, p-AKT (Ser473), total AKT, H3, and GAPDH. Loss of the G9a-CtBP2 interaction (G9a^ΔPre-SET^, Δ885–893) increases PTEN expression and reduces AKT phosphorylation. *H*, working model: G9a cooperates with CtBP2 to deposit H3K9me2 at the PTEN promoter, repressing PTEN transcription and thereby maintaining PI3K–AKT signaling and proliferation of HCT116 cells.

To elucidate the molecular programs governed by the CtBP2-G9a axis, we performed transcriptome profiling of HCT116 cells subjected to G9a depletion and reconstituted with either shRNA-resistant G9a^WT^ or the binding-deficient G9a^ΔPre-SET^, with shNC-transduced cells serving as a control. Differential expression analysis identified 1020 genes whose expression changes upon G9a depletion were restored by G9a^WT^ reconstitution (Fig. S5*A*). Comparative analyses further refined this set to 126 genes whose regulation strictly required an intact G9a-CtBP2 interface (Fig. 6*A*), underscoring the functional necessity of their physical interaction. KEGG pathway enrichment analysis revealed significant enrichment of oncogenic pathways such as PI3K-AKT, Fanconi anemia, and FoxO signaling, all closely associated with colorectal cancer progression and therapeutic resistance (Fig. 6*B*). Collectively, these results demonstrate that disruption of the CtBP2-G9a interaction rewires transcriptional programs critical for malignant growth, thereby directly linking the structural interface to oncogenic signaling networks.

Among the interface-dependent targets, we focused on PTEN, a canonical tumor suppressor that restrains PI3K-AKT signaling (57, 58, 59). Transcriptome analyses revealed that depletion of G9a increased PTEN mRNA, whereas reconstitution with G9a^WT^—but not the binding-deficient G9a^ΔPre-SET^—restored repression (Fig. 6, *C* and *G*). Chromatin immunoprecipitation (ChIP) assays further demonstrated that loss of G9a reduced H3K9me2 enrichment at the PTEN promoter, a defect that was efficiently rescued by G9a^WT^ but not G9a^ΔPre-SET^ (Fig. 6*D*). Consistently, FLAG-G9a ChIP showed diminished recruitment of G9a to the PTEN promoter upon disruption of its interaction with CtBP2 (Fig. 6*E*), indicating that CtBP2 facilitates G9a recruitment to this locus. To validate the specificity of these observations, we next assessed whether H3K27me3 deposition at the PTEN promoter was influenced by the CtBP2-G9a interaction. ChIP–qPCR analyses revealed that H3K27me3 showed negligible enrichment at the PTEN promoter under all tested conditions, including G9a depletion or reconstitution with either G9a^WT^ or G9a^ΔPre-SET^ in G9a-knockdown HCT116 cells (Fig. 6*F*). These findings establish that PTEN repression is specifically mediated by G9a-dependent H3K9me2 deposition, which in turn relies on its interaction with CtBP2. Thus, at the signaling level, disruption of the G9a-CtBP2 interface led to elevated PTEN abundance accompanied by reduced AKT phosphorylation (Fig. 6*G*), thereby attenuating downstream PI3K-AKT activity.

Finally, to further determine whether CtBP2 oligomerization is required for potentiating G9a(WT)-mediated transcriptional repression—particularly with regard to PTEN expression, H3K9me2 deposition, and downstream PTEN-AKT signaling—we performed rescue experiments in CtBP2-knockdown HCT116 cells to eliminate interference from endogenous CtBP2. Reintroduction of wild-type oligomeric CtBP2, but not the oligomerization-defective CtBP2^Monomer^ mutant, restored PTEN repression, reinstated AKT phosphorylation, and recovered H3K9me2 enrichment at the PTEN promoter (Fig. S6, *A* and B). These data further demonstrate that only oligomeric CtBP2 is competent to cooperate with endogenous G9a to drive PTEN silencing through H3K9me2 deposition. Collectively, these results support a mechanistic model in which CtBP2 recruits G9a to the PTEN promoter to enforce H3K9me2 deposition and transcriptional silencing. Disruption of CtBP2 oligomerization, or of the CtBP2–G9a interface, compromises G9a recruitment and repressive histone methylation, thereby restoring PTEN expression and dampening PI3K-AKT activity. Ultimately, this suppresses colorectal cancer cell proliferation (Fig. 6*H*).

## Discussion

Our structural and biochemical analyses suggest a molecular mechanism that may couple cellular redox status to facultative heterochromatin formation in colorectal cancer (CRC). Specifically, we show that NAD(H)-biased conditions stabilize tetrameric CtBP1/2, which in turn engages a unique pre-SET motif present only in G9a (but not in GLP) to enhance its catalytic activity. Although we did not quantify the magnitude of this effect, our assays consistently indicate that CtBP1/2 oligomerization stimulates G9a methyltransferase function. The consequent increase in H3K9me2 deposition facilitates repression of tumor-suppressor genes exemplified by PTEN, thereby sustaining PI3K-AKT signaling and malignant proliferation (Fig. 6*H*). Importantly, disruption of this protein-protein interface—either by genetic deletion of the G9a pre-SET motif or by CtBP2 monomerization—abolished CtBP2-mediated stimulation of G9a, restored PTEN expression, and impaired tumor cell fitness, consistent with our functional data. Together, these findings identify the CtBP1/2-G9a complex as a druggable metabolic–epigenetic node whose activity is likely tuned by the intracellular NADH/NAD^+^ balance, integrating metabolic state with chromatin-mediated transcriptional repression.

Although CtBP1/2 have long been implicated in association with “G9a/GLP” complexes, whether these interactions are direct and how they are mediated has remained unclear. Our data refine this view by demonstrating paralog-specific engagement through mutually exclusive interfaces: G9a is recognized *via* the DTAWDLTPE motif (residues 885–893) within its pre-SET domain, whereas GLP binds CtBP1/2 through a distinct DLS element in its Glu-Cys region (Figs. 3, *D*–*F* and S3). While our study does not directly dissect GLP- *versus* G9a-dependent functions, previous work has highlighted that the two paralogs have non-redundant roles in chromatin regulation (60, 61). This paralog-specific recognition mechanism therefore suggests a therapeutic opportunity: selectively disrupting the CtBP2-G9a interface could attenuate oncogenic transcriptional programs driven by G9a, while sparing GLP-mediated epigenetic regulation. Such an approach may offer advantages over small-molecule catalytic inhibitors of G9a/GLP, which inactivate both paralogs indiscriminately and are associated with widespread epigenetic perturbation and toxicity.

Previous genetic studies established that CtBP dimerization or higher-order oligomerization is required for transcriptional repression (8, 14, 19), yet the underlying molecular basis has remained unclear. Our structural and biochemical analyses suggest that tetramerization promotes productive engagement of G9a, likely by increasing its local concentration and stabilizing a complex that enhances methyltransferase activity without altering substrate specificity. This requirement for tetrameric CtBP offers a mechanistic rationale for why oligomerization-defective mutations act as dominant-negative alleles in *Drosophila* (8), and why NAD(H) mimetics that promote CtBP tetramerization have been shown to reinforce transcriptional repression. More broadly, our findings support an emerging principle in chromatin regulation: oligomeric corepressors such as PRC2, LSD1-CoREST, and Sin3A exploit multivalency to assemble catalytically competent chromatin-modifying complexes.

Elevated levels of G9a and CtBP2 are each associated with poor prognosis in colorectal cancer (47, 48, 49, 53), and our data now suggest that they cooperate functionally at the chromatin level. Specifically, we demonstrate co-occupancy at the PTEN promoter, interface-dependent H3K9me2 deposition, and supra-additive growth inhibition upon dual knockdown. Importantly, a catalytically competent G9a mutant that is unable to bind CtBP2 only partially restores proliferation, indicating that their physical interaction—rather than the mere presence of both proteins—is critical for maximal oncogenic output. From a therapeutic perspective, the pre-SET pocket is surface-exposed and relatively shallow, making it an attractive target for peptidomimetic or fragment-based inhibitor design. These findings open the door to therapeutic exploration: a cell-penetrant pre-SET–derived peptide (residues 881–893) offers a potential strategy to competitively disrupt the CtBP2–G9a complex, restore PTEN expression, and reduce CRC growth, warranting further investigation as a selective epigenetic intervention.

Certain limitations of our study should be acknowledged. First, the crystal structure was determined using a truncated CtBP2 fragment (residues 31–364) in complex with a G9a pre-SET motif peptide. While this construct faithfully recapitulates the binding interface, we cannot exclude additional regulatory contributions from the disordered N- and C-terminal regions. Moreover, since G9a generally functions as a homodimer or heterodimer, it remains unclear how CtBP2 engages dimeric G9a and whether such higher-order assemblies impose distinct structural or functional consequences—questions that could be addressed in future work by cryo-electron microscopy. Second, we have not directly quantified how physiological shifts in the NAD^+^/NADH ratio influence the CtBP2-G9a interaction in live cells; approaches such as NAD(H) biosensors will be required to rigorously address this. Finally, given that acyl-CoA metabolites have been reported to disrupt CtBP2 oligomerization (20, 21, 22), it will be of considerable interest to determine whether acyl-CoAs similarly regulate G9a activity through CtBP2, thereby linking lipid metabolism to chromatin regulation. Addressing these open questions will not only refine the molecular model proposed here but also broaden our understanding of metabolic control of epigenetic programs in cancer.

## Experimental procedures

### Cell cultures

HEK293T cells were cultured in Dulbecco’s Modified Eagle Medium (DMEM) supplemented with 10% (v/v) fetal bovine serum (AMOBIO, FBSAB001) and 1% penicillin-streptomycin (Pricella, PB180120). Human colorectal cancer HCT-116 cells were maintained in RPMI-1640 medium supplemented with 10% (v/v) fetal bovine serum and 1% penicillin–streptomycin. All cells were grown at 37 °C in a humidified incubator with 5% CO_2_.

### Expression and purification of recombinant human CtBPs

Human CtBP cDNAs were subcloned into the pET-28a-SUMO vector to generate N-terminal His_6_-SUMO fusion proteins. All plasmids were verified by Sanger sequencing (Sangon Biotech, Shanghai) and transformed into *E. coli* Rosetta 2 (DE3) cells for recombinant protein expression. Protein production was induced with 0.4 mM isopropyl-β-D-thiogalactopyranoside (IPTG) at an OD_600_ of ∼0.8, followed by incubation at 20 °C for 20 h. Harvested cells were resuspended in lysis buffer [50 mM Tris-HCl (pH 8.0), 500 mM NaCl, 5% (v/v) glycerol, 1 mM phenylmethylsulfonyl fluoride, and 2 mM β-mercaptoethanol] and disrupted by ultrasonication. Insoluble material was removed by centrifugation, and the clarified supernatant was loaded onto Ni-NTA affinity resin (GenScript, L00666–100). Bound proteins were eluted with lysis buffer containing 300 mM imidazole. The His_6_-SUMO tag was cleaved using SUMO protease, and tag-free CtBP2_31-364_ was further purified by size-exclusion chromatography on a HiLoad 16/600 Superdex 200 pg column (Cytiva) equilibrated with buffer [50 mM Tris-HCl (pH 8.0), 150 mM NaCl, and 2 mM DTT]. Peak fractions were pooled, concentrated, flash-frozen in liquid nitrogen, and stored at −80 °C. Protein purity was assessed by SDS-PAGE. For crystallization experiments, the proteins underwent additional polishing by ion-exchange chromatography (Capto HiRes Q 5/50) followed by gel filtration on a Superose 6 Increase 10/300 Gl column.

### FLAG pull-down assays

FLAG pull-down assays were performed at 4 °C in pull-down buffer containing 50 mM Tris-HCl (pH 8.0), 150 mM NaCl, 2 mM DTT, and 0.1% NP-40. SUMO-G9a constructs (residues 1–260, 261–630, 631–880, 881–1210, 881–913, 913–1193, 1193–1210, and 893–913) fused to a FLAG tag were expressed in *E. coli* Rosetta 2 (DE3) and purified *via* affinity chromatography. The FLAG-tagged proteins were immobilized on Anti-FLAG Resin (GenScript, L00432-5) and washed three times with pull-down buffer. Resin-bound proteins were then incubated with full-length SUMO-CtBP2 for 2 h at 4 °C with gentle agitation. Following three additional washes, bound complexes were eluted using FLAG peptide and analyzed by SDS-PAGE. All SUMO-G9a_881-913_ mutants were generated by PCR-based site-directed mutagenesis and assayed under identical FLAG pull-down conditions. Pull-down assays using StrepI- or MBP-tagged proteins were performed following the same procedure, employing the respective affinity resins.

### Protein crystallization, data collection, and structure determination

CtBP2_31-364_ was mixed with the G9a_881-893_ peptide (1:5 M ratio) on ice for 2 h, then crystallized using the sitting-drop vapor diffusion method at 18 °C with 1 μl of protein and 1 μl of reservoir solution (0.1 M HEPES pH 7.5, 6% PVP, 30% pentaerythritol ethoxylate). The diffraction data of the crystals were collected at beamlines BL02U1 and BL19U1 of Shanghai Synchrotron Radiation Facility (SSRF) at 100 K. Intensity data were integrated and scaled by HKL2000 (62). Processing of X-ray diffraction data collected in oscillation mode (62) or XDS (https://doi.org/10.1107/S0907444909047337). The complex structures were solved by the molecular replacement method by Phaser (63) using the structure of CtBP2 (PDB ID: 8ATI) as the searching model (18). Manual model building and refinement were carried out iteratively using Coot (64) and Refmac5 (65). The final models were validated by MolProbity (66) and statistics are summarized in Table S3. The figures were produced using PyMOL (http://www.pymol.org/).

### Isothermal titration calorimetry (ITC) measurements

ITC experiments were performed on a MicroCal iTC200 instrument (Malvern) at 25 °C. All proteins and peptides were prepared in gel filtration buffer containing 20 mM Tris-HCl (pH 8.0) and 100 mM NaCl. For each titration, CtBP2_31-364_-StrepI at concentrations of 2.0 mM was titrated into a sample cell containing 25 μM peptide. A total of 20 injections were applied, with the initial injection of 1 μl followed by 19 injections of 2 μl each. Data were analyzed using Origin 2016 software with a one-site binding model.

### Co-immunoprecipitation (Co-IP) and Western blotting

Plasmids encoding pCDNA3.1-CtBP2-(1-445)-FLAG, pCDNA3.1-GLP-(1-1298)^ΔPre-SET^-Myc, pCDNA3.1-GLP-(1-1298)-Myc, pCDNA3.1-G9a-(1-1210)^ΔPre-SET^-Myc, and pCDNA3.1-G9a-(1-1210)-Myc were transfected individually or in combination into HEK293T cells using PEI 40,000 (YEASEN, 40816ES01) according to the manufacturer’s instructions. Forty-eight hours post-transfection, cells were harvested and lysed in buffer containing 50 mM HEPES (pH 7.9), 150 mM NaCl, 10% glycerol, 0.1% NP-40, 1.5 mM MgCl_2_, and 2 mM DTT, supplemented with protease inhibitors. Cell lysates were sonicated and centrifuged at 12,000 rpm for 30 min at 4 °C. The resulting supernatants were incubated with Anti-FLAG Resin for 2 h at 4 °C. Beads were washed three times with buffer containing 50 mM Tris-HCl (pH 8.0), 150 mM NaCl, 2 mM DTT, and 0.1% NP-40. Bound proteins were eluted using FLAG peptide at 4 °C, and the presence of FLAG- and MYC-tagged proteins was analyzed by Western blotting with epitope-specific antibodies.

### Histone methyltransferase assay

Histone methyltransferase activity was evaluated by incubating increasing amounts of CtBP2 oligomer or monomer (0, 1, 2, or 4 μg) with 8 μg of G9a (641–1193)^WT^ or G9a (641–1193)^ΔPre-SET^, 0.25 μg of histone H3, and 32 μM S-adenosylmethionine (SAM) in a 20 μl reaction. Reactions were conducted in buffer containing 50 mM Tris (pH 8.0), 50 mM NaCl, 2.5 mM MgCl_2_, 1 mM EDTA, and 2.5 mM DTT at 30 °C for 4 h. Reactions were terminated by adding 10 μl of 4× SDS loading buffer and heating at 85 °C for 5 min. Proteins were separated by SDS-PAGE and analyzed by Western blotting with an anti-H3K9me2 antibody to detect dimethylation at histone H3 lysine 9.

### Luciferase reporter assay

A previously established UHRF1-luciferase reporter system (kindly provided by Hai-Ning Du, Wuhan University) was employed to assess G9a activity in HEK293T cells (44). Cells were co-transfected with 0.2 μg of the 5×GAL4-UHRF1-luciferase reporter, 2 μg of GAL4-HA-G9a (wild-type or ΔPre-SET mutant) or the GAL4-HA empty vector as control, and 0.5 μg of pcDNA3.0-Renilla, with or without 1 μg of pcDNA3.1-CtBP2 or its control vector. After 48 h, luciferase activity was measured using the Dual-Luciferase Reporter Assay Kit (Vazyme, DL101-01) according to the manufacturer’s instructions. Renilla luciferase was used as an internal control to normalize for transfection efficiency.

### Analytical gel filtration

Size-exclusion chromatography (SEC) was employed to examine the oligomeric states of human CtBP2, including the wild-type oligomer and a monomeric mutant variant (R147A, R148A, R169A, and R177A). Chromatography was performed on a Superose 6 Increase 10/300 Gl column equilibrated with SEC buffer (20 mM Tris-HCl, pH 8.0; 150 mM NaCl; 2 mM DTT). Protein samples were applied to the column and eluted at a flow rate of 0.35 ml/min, with elution profiles monitored by absorbance at 280 nm.

### CtBP2 oligomerization Co-IP assay

HEK293 T cells were used to examine CtBP2 oligomerization. Full-length CtBP2 cDNA (wild-type or mutant) was subcloned into the pCDNA3.1 vector with either a C-terminal FLAG or Myc epitope tag, generating CtBP2-FLAG and CtBP2-Myc constructs. Cells were co-transfected with matched pairs of CtBP2-FLAG and CtBP2-Myc plasmids (WT/WT or mutant/mutant). Forty-eight hours after transfection, cells were harvested and lysed in buffer containing 50 mM HEPES (pH 7.9), 150 mM NaCl, 10% glycerol, 0.1% NP-40, 1.5 mM MgCl_2_, and 2 mM DTT, supplemented with protease inhibitors. Lysates were sonicated and clarified by centrifugation at 12,000 rpm for 30 min at 4 °C. The supernatants were incubated with Anti-FLAG Resin for 2 h at 4 °C, followed by three washes with buffer containing 50 mM Tris-HCl (pH 8.0), 150 mM NaCl, 2 mM DTT, and 0.1% NP-40. Bound proteins were eluted with FLAG peptide at 4 °C and analyzed by Western blotting using anti-FLAG and anti-Myc antibodies. Differences in Myc signal intensity co-precipitated with FLAG-tagged CtBP2 reflected the relative oligomerization capacity of WT *versus* mutant CtBP2 proteins.

### Lentivirus production and stable cell line generation

Lentiviral particles were produced in HEK293 T cells by co-transfecting the packaging plasmids psPAX2 and pVSV-G with either pCDH-EF1-3×Flag-empty, pCDH-EF1-3×Flag-G9a (1–1210)^WT^, or pCDH-EF1-3×Flag-G9a (1–1210)^ΔPre-SET^. Transfections were performed using PEI with 2.25 μg psPAX2, 0.75 μg pVSV-G, and 3 μg of the respective expression construct per dish. Forty-eight hours after transfection, viral supernatants were collected, clarified by centrifugation, and used to infect HCT116 cells. Stable cell populations were established by puromycin selection, initiated 48 h post-infection.

### Construction of knockdown cell lines

Lentiviruses carrying pLKO.1-shG9a#1, pLKO.1-shG9a#2, pLKO.1-shCtBP2#1, pLKO.1-shCtBP2#2, or the control vector pLKO.1-shNC were produced as described above. Viral supernatants were harvested and used to infect HCT116 cells. At 48 h post-infection, cells were subjected to puromycin selection to generate stable knockdown populations targeting G9a or CtBP2. Knockdown efficiency was validated by Western blotting.

### Cell proliferation and colony formation assays

shG9a-resistant G9a (1–1210)^WT^ and G9a (1–1210)^ΔPre-SET^ cDNAs were individually cloned into the pCDH-EF1-3 × Flag vector. HCT116 cells with endogenous G9a depletion were transfected with either pCDH-EF1-3 × Flag-G9a (1–1210)^WT^ or pCDH-EF1-3 × Flag-G9a (1–1210)^ΔPre-SET^, yielding cell lines with comparable expression of wild-type or mutant G9a. Alongside shNC and G9a-knockdown HCT116 controls, these engineered cell lines were subjected to proliferation and colony formation assays. For proliferation assays, 1500 cells per well were seeded into 96-well plates, and cell growth was monitored using the Cell Counting Kit-8 (CCK-8). At the indicated time points, CCK-8 reagent was added to each well, incubated for 1 h at 37 °C, and absorbance at 450 nm was measured. Wells containing medium alone served as blanks. For colony formation assays, 1000 cells were plated into 12-well plates and cultured for approximately 10 days. Colonies were then washed twice with PBS, stained with 0.05% Crystal violet, air-dried, and imaged for quantification.

### RNA sequencing

Total RNA was isolated from HCT116-shNC, HCT116-shG9a, and HCT116-shG9a cells reconstituted with either full-length G9a or the ΔPre-SET mutant. In the rescued lines, G9a expression was restored to levels comparable to those in HCT116-shNC cells. RNA extraction was performed using TRIzol reagent according to the manufacturer’s instructions. Library construction and sequencing were carried out by Sangon Biotech. Differentially expressed genes (DEGs) were defined by a threshold of |log_2_(fold change)| ≥ 1.5 with a *p* value < 0.05.

### Real-time quantitative PCR (RT-qPCR)

Total RNA was isolated using the AG RNAex Pro reagent (Accurate Biology, AG21101) and reverse-transcribed with the Evo M-MLV Reverse Transcriptase Kit (Accurate Biology, AG11728). Quantitative PCR was performed with the SYBR Green Pro Taq HS qPCR Kit (Accurate Biology, 1G11739) in 96-well clear plates (0.1 ml; Accurate Biology, AG12116) on an AriaMx Real-Time PCR System. Relative transcript levels were calculated using the comparative ΔΔCt method, with GAPDH as the normalization control. All reactions were conducted in triplicate. Primer sequences used for amplification are listed in Table S1.

### ChIP-qPCR

Chromatin immunoprecipitation (ChIP) assays were performed as previously described with minor modifications (67). Briefly, HCT116 cells grown in 15-cm dishes were washed with 1 × PBS and cross-linked with 1% formaldehyde (Thermo Scientific, Cat#28908) for 10 min at room temperature with gentle rocking, followed by quenching with 125 mM glycine for 5 min. Cross-linked cells were washed once with cold PBS, collected by centrifugation, and lysed in ChIP SDS lysis buffer (50 mM Tris, pH 8.0; 10 mM EDTA; 1% SDS; 1 mM DTT; protease inhibitors). Crude nuclei were pelleted at 2000 rpm for 5 min at 4 °C and resuspended in sonication buffer (50 mM Tris-HCl, pH 8.0; 1% SDS; 10 mM EDTA; 1 mM DTT; protease inhibitors). Chromatin was sonicated at 35% amplitude (5 s on/10 s off, 25–30 cycles) to yield fragments of 200 to 500 bp. Soluble chromatin was diluted with ChIP dilution buffer (50 mM Tris-HCl, pH 8.0; 0.5% Triton X-100; 5 mM EDTA; 150 mM NaCl; 1 mM DTT; protease inhibitors) to reduce the final SDS concentration to 0.1%, and centrifuged at 13,000*g* for 15 min at 4 °C, with 1% of the supernatant reserved as input. For immunoprecipitation, 100 to 200 μg of chromatin was incubated overnight at 4 °C with 5 μg of anti-H3K27me3, anti-H3K9me2, anti-FLAG, or control IgG antibodies. Protein A/G magnetic beads (50 μl; MCE, Cat#HY-K0202), pre-blocked overnight with 0.5% BSA in ChIP dilution buffer, were added to capture the antibody-chromatin complexes. Beads were sequentially washed three times with low-salt buffer, once with high-salt buffer, once with LiCl buffer, and twice with TE buffer. Bound chromatin was eluted using elution buffer (50 mM Tris, pH 8.0; 10 mM EDTA; 1% SDS) and reverse cross-linked at 65 °C overnight. Samples were then treated with proteinase K and RNase A, and DNA was purified by phenol:chloroform:isoamyl alcohol extraction. Enrichment of H3K27me3, H3K9me2, and FLAG-G9a was determined by qPCR using gene-specific primers (Table S2) (50). All experiments were independently repeated at least three times.

### Quantification and statistical analysis

All statistical evaluations were performed using GraphPad Prism 8. For datasets involving more than two groups, one-way ANOVA followed by Tukey’s *post hoc* multiple comparison test was applied. When analyses involved two independent variables, two-way ANOVA with Tukey’s *post hoc* test was conducted. All statistical tests were two-tailed. Data are presented as mean ± SD, and significance was defined as *p* < 0.05 (∗*p* < 0.05, ∗∗*p* < 0.01, ∗∗∗*p* < 0.001).

### Data and code availability

The RNA sequencing datasets generated in this study have been deposited in the Sequence Read Archive (SRA). Accession numbers are provided in the Key Resources Table.

## Supporting information

This article contains supporting information.

## Conflict of interest

The authors declare that they do not have any conflicts of interest with the content of this article.

## References

[1] Hildebrand J.D., Soriano P. (2002). Overlapping and unique roles for C-terminal binding protein 1 (CtBP1) and CtBP2 during mouse development. Mol. Cell Biol..

[2] Strickland B.A., Babl A., Wolff L., Singh P., Friano M.E., Greulich F. (2025). C-terminal binding protein 2 interacts with JUNB to control macrophage inflammation. Life Sci. Alliance.

[3] Bi C.L., Cheng Q., Yan L.Y., Wu H.Y., Wang Q., Wang P. (2022). A prominent gene activation role for C-terminal binding protein in mediating PcG/trxG proteins through Hox gene regulation. Development.

[4] Ray S.K., Li H.J., Metzger E., Schüle R., Leiter A.B. (2014). CtBP and associated LSD1 are required for transcriptional activation by NeuroD1 in gastrointestinal endocrine cells. Mol. Cell Biol..

[5] Kim T.W., Kang B.H., Jang H., Kwak S., Shin J., Kim H. (2015). Ctbp2 modulates NuRD-Mediated deacetylation of H3K27 and facilitates PRC2-Mediated H3K27me3 in active embryonic stem cell genes during exit from pluripotency. Stem Cells.

[6] Chinnadurai G. (2007). Transcriptional regulation by C-terminal binding proteins. Int. J. Biochem. Cell Biol..

[7] Shi Y., Sawada J.I., Sui G., Affar E.B., Whetstine J.R., Lan F. (2003). Coordinated histone modifications mediated by a CtBP co-repressor complex. Nature.

[8] Bhambhani C., Chang J.L., Akey D.L., Cadigan K.M. (2011). The oligomeric state of CtBP determines its role as a transcriptional co-activator and co-repressor of Wingless targets. EMBO J..

[9] Tachibana M., Ueda J., Fukuda M., Takeda N., Ohta T., Iwanari H. (2005). Histone methyltransferases G9a and GLP form heteromeric complexes and are both crucial for methylation of euchromatin at H3-K9. Genes Dev..

[10] Shi Y., Lan F., Matson C., Mulligan P., Whetstine J.R., Cole P.A. (2004). Histone demethylation mediated by the nuclear amine oxidase homolog LSD1. Cell.

[11] Eshelman M.A., Shah M., Raup-Konsavage W.M., Rennoll S.A., Yochum G.S. (2017). TCF7L1 recruits CtBP and HDAC1 to repress DICKKOPF4 gene expression in human colorectal cancer cells. Biochem. Biophys. Res. Commun..

[12] Dressel U., Bailey P.J., Wang S.C., Downes M., Evans R.M., Muscat G.E. (2001). A dynamic role for HDAC7 in MEF2-mediated muscle differentiation. J. Biol. Chem..

[13] Zhang C.L., McKinsey T.A., Lu J.R., Olson E.N. (2001). Association of COOH-terminal-binding protein (CtBP) and MEF2-interacting transcription repressor (MITR) contributes to transcriptional repression of the MEF2 transcription factor. J. Biol. Chem..

[14] Kuppuswamy M., Vijayalingam S., Zhao L.J., Zhou Y., Subramanian T., Ryerse J., Chinnadurai G. (2008). Role of the PLDLS-binding cleft region of CtBP1 in recruitment of core and auxiliary components of the corepressor complex. Mol. Cell Biol..

[15] Chan K.L., Gomez J., Cardinez C., Kumari N., Sparbier C.E., Lam E.Y.N. (2022). Inhibition of the CtBP complex and FBXO11 enhances MHC class II expression and anti-cancer immune responses. Cancer Cell.

[16] Nichols J.C., Schiffer C.A., Royer W.E. (2021). NAD(H) phosphates mediate tetramer assembly of human C-terminal binding protein (CtBP). J. Biol. Chem..

[17] Bellesis A.G., Jecrois A.M., Hayes J.A., Schiffer C.A., Royer W.E. (2018). Assembly of human C-terminal binding protein (CtBP) into tetramers. J. Biol. Chem..

[18] Goradia N., Werner S., Mullapudi E., Greimeier S., Bergmann L., Lang A. (2024). Master corepressor inactivation through multivalent SLiM-induced polymerization mediated by the oncogene suppressor RAI2. Nat. Commun..

[19] Thio S.S., Bonventre J.V., Hsu S.I. (2004). The CtBP2 co-repressor is regulated by NADH-dependent dimerization and possesses a novel N-terminal repression domain. Nucleic Acids Res..

[20] Sekiya M., Ma Y., Kainoh K., Saito K., Yamazaki D., Tsuyuzaki T. (2023). Loss of CtBP2 may be a mechanistic link between metabolic derangements and progressive impairment of pancreatic beta cell function. Cell Rep..

[21] Sekiya M., Kainoh K., Sugasawa T., Yoshino R., Hirokawa T., Tokiwa H. (2021). The transcriptional corepressor CtBP2 serves as a metabolite sensor orchestrating hepatic glucose and lipid homeostasis. Nat. Commun..

[22] Saito K., Sekiya M., Kainoh K., Yoshino R., Hayashi A., Han S.I. (2023). Obesity-induced metabolic imbalance allosterically modulates CtBP2 to inhibit PPAR-alpha transcriptional activity. J. Biol. Chem..

[23] Sekiya M., Kainoh K., Saito K., Yamazaki D., Tsuyuzaki T., Chen W. (2024). C-Terminal binding protein 2 emerges as a critical player linking metabolic imbalance to the pathogenesis of obesity. J. Atheroscler. Thromb..

[24] Tachibana M., Sugimoto K., Nozaki M., Ueda J., Ohta T., Ohki M. (2002). G9a histone methyltransferase plays a dominant role in euchromatic histone H3 lysine 9 methylation and is essential for early embryogenesis. Genes Dev..

[25] Shinkai Y., Tachibana M. (2011). H3K9 methyltransferase G9a and the related molecule GLP. Genes Dev..

[26] Sanchez N.A., Kallweit L.M., Trnka M.J., Clemmer C.L., Al-Sady B. (2021). Heterodimerization of H3K9 histone methyltransferases G9a and GLP activates methyl reading and writing capabilities. J. Biol. Chem..

[27] Zhang T., Termanis A., Özkan B., Bao X.X., Culley J., de Lima Alves F. (2016). G9a/GLP complex maintains imprinted DNA methylation in embryonic stem cells. Cell Rep..

[28] Tachibana M., Matsumura Y., Fukuda M., Kimura H., Shinkai Y. (2008). G9a/GLP complexes independently mediate H3K9 and DNA methylation to silence transcription. EMBO J..

[29] Ueda J., Tachibana M., Ikura T., Shinkai Y. (2006). Zinc finger protein wiz links G9a/GLP histone methyltransferases to the co-repressor molecule CtBP. J. Biol. Chem..

[30] Nishio H., Walsh M.J. (2004). CCAAT displacement protein/cut homolog recruits G9a histone lysine methyltransferase to repress transcription. Proc. Natl. Acad. Sci. U. S. A..

[31] Ogawa H., Ishiguro K.I., Gaubatz S., Livingston D.M., Nakatani Y. (2002). A complex with chromatin modifiers that occupies E2F- and Myc-responsive genes in G0 cells. Science.

[32] Shankar S.R., Bahirvani A.G., Rao V.K., Bharathy N., Ow J.R., Taneja R. (2013). G9a, a multipotent regulator of gene expression. Epigenetics.

[33] Fong K.W., Zhao J.C., Lu X., Kim J., Piunti A., Shilatifard A. (2022). PALI1 promotes tumor growth through competitive recruitment of PRC2 to G9A-target chromatin for dual epigenetic silencing. Mol. Cell.

[34] Poulard C., Noureddine L.M., Pruvost L., Le Romancer M. (2021). Structure, activity, and function of the protein lysine methyltransferase G9a. Life (Basel).

[35] Krishnan S., Horowitz S., Trievel R.C. (2011). Structure and function of histone H3 lysine 9 methyltransferases and demethylases. Chembiochem.

[36] Ni Y., Shi M., Liu L., Lin D., Zeng H., Ong C. (2024). G9a in cancer: mechanisms, therapeutic advancements, and clinical implications. Cancers (Basel).

[37] Hajar M., Werner T., Gajic M., Stark H., Sadek B. (2025). Targeting histone H3K9 methyltransferase G9a as a potential therapeutic strategy for neuropsychiatric disorders. Med. Res. Rev..

[38] Raicu A.M., Suresh M., Arnosti D.N. (2024). A regulatory role for the unstructured C-terminal domain of the CtBP transcriptional corepressor. J. Biol. Chem..

[39] Raicu A.M., Kadiyala D., Niblock M., Jain A., Yang Y., Bird K.M. (2023). The cynosure of CtBP: evolution of a bilaterian transcriptional corepressor. Mol. Biol. Evol..

[40] Bergman L.M., Blaydes J.P. (2006). C-terminal binding proteins: emerging roles in cell survival and tumorigenesis. Apoptosis.

[41] Silva-Carvalho A.E., Filiú-Braga L.D.C., Bogéa G.M.R., de Assis A.J.B., Pittella-Silva F., Saldanha-Araujo F. (2024). GLP and G9a histone methyltransferases as potential therapeutic targets for lymphoid neoplasms. Cancer Cell Int..

[42] Pang K.K.L., Sharma M., Sajikumar S. (2019). Epigenetics and memory: emerging role of histone lysine methyltransferase G9a/GLP complex as bidirectional regulator of synaptic plasticity. Neurobiol. Learn Mem..

[43] Jecrois A.M., Dcona M.M., Deng X., Bandyopadhyay D., Grossman S.R., Schiffer C.A. (2021). Cryo-EM structure of CtBP2 confirms tetrameric architecture. Structure.

[44] Geng Q., Kong Y.Y., Li W., Zhang J., Ma H., Zhang Y. (2023). Dynamic phosphorylation of G9a regulates its repressive activity on chromatin accessibility and mitotic progression. Adv. Sci. (Weinh).

[45] Kumar V., Carlson J.E., Ohgi K.A., Edwards T.A., Rose D.W., Escalante C.R. (2002). Transcription corepressor CtBP is an NAD(+)-regulated dehydrogenase. Mol. Cell.

[46] Zhao L.J., Kuppuswamy M., Vijayalingam S., Chinnadurai G. (2009). Interaction of ZEB and histone deacetylase with the PLDLS-binding cleft region of monomeric C-terminal binding protein 2. BMC Mol. Biol..

[47] Bergin C.J., Zouggar A., Mendes da Silva A., Fenouil T., Haebe J.R., Masibag A.N. (2024). The dopamine transporter antagonist vanoxerine inhibits G9a and suppresses cancer stem cell functions in colon tumors. Nat. Cancer.

[48] Bergin C.J., Zouggar A., Haebe J.R., Masibag A.N., Desrochers F.M., Reilley S.Y. (2021). G9a controls pluripotent-like identity and tumor-initiating function in human colorectal cancer. Oncogene.

[49] Qin J., Zeng Z., Luo T., Li Q., Hao Y., Chen L. (2018). Clinicopathological significance of G9A expression in colorectal carcinoma. Oncol. Lett..

[50] Bhat A.V., Palanichamy Kala M., Rao V.K., Pignata L., Lim H.J., Suriyamurthy S. (2019). Epigenetic regulation of the PTEN-AKT-RAC1 axis by G9a is critical for tumor growth in alveolar Rhabdomyosarcoma. Cancer Res..

[51] Fenech K., Micallef I., Baron B. (2024). 5-Fluorouracil dose escalation generated desensitized colorectal cancer cells with reduced expression of protein methyltransferases and no epithelial-to-mesenchymal transition potential. Oncol. Res..

[52] Paschall A.V., Yang D., Lu C., Choi J.H., Li X., Liu F. (2015). H3K9 trimethylation silences fas expression to confer Colon carcinoma immune escape and 5-Fluorouracil chemoresistance. J. Immunol..

[53] de Barrios O., Győrffy B., Fernández-Aceñero M.J., Sánchez-Tilló E., Sánchez-Moral L., Siles L. (2017). ZEB1-induced tumourigenesis requires senescence inhibition *via* activation of DKK1/mutant p53/Mdm2/CtBP and repression of macroH2A1. Gut.

[54] Erlandsen H., Jecrois A.M., Nichols J.C., Cole J.L., Royer W.E. (2022). NADH/NAD(+) binding and linked tetrameric assembly of the oncogenic transcription factors CtBP1 and CtBP2. FEBS Lett..

[55] Meng Y., Ding J., Wang Y., Wang J., Huang W., Jiang W. (2025). The transcriptional repressor Ctbp2 as a metabolite sensor regulating cardiomyocytes proliferation and heart regeneration. Mol. Med..

[56] He Y., He Z., Lin J., Chen C., Chen Y., Liu S. (2021). CtBP1/2 differentially regulate genomic stability and DNA repair pathway in high-grade serous ovarian cancer cell. Oncogenesis.

[57] Chin Y.R., Yuan X., Balk S.P., Toker A. (2014). PTEN-deficient tumors depend on AKT2 for maintenance and survival. Cancer Discov..

[58] Haddadi N., Lin Y., Travis G., Simpson A.M., Nassif N.T., McGowan E.M. (2018). PTEN/PTENP1: 'regulating the regulator of RTK-dependent PI3K/Akt signalling', new targets for cancer therapy. Mol. Cancer.

[59] Salmena L., Carracedo A., Pandolfi P.P. (2008). Tenets of PTEN tumor suppression. Cell.

[60] Link P.A., Gangisetty O., James S.R., Woloszynska-Read A., Tachibana M., Shinkai Y. (2009). Distinct roles for histone methyltransferases G9a and GLP in cancer germ-line antigen gene regulation in human cancer cells and murine embryonic stem cells. Mol. Cancer Res..

[61] Battisti V., Pontis J., Boyarchuk E., Fritsch L., Robin P., Ait-Si-Ali S. (2016). Unexpected distinct roles of the related histone H3 lysine 9 methyltransferases G9a and G9a-Like protein in myoblasts. J. Mol. Biol..

[62] Otwinowski Z., Minor W. (1997). Processing of X-ray diffraction data collected in oscillation mode. Methods Enzymol..

[63] McCoy A.J., Grosse-Kunstleve R.W., Adams P.D., Winn M.D., Storoni L.C., Read R.J. (2007). Phaser crystallographic software. J. Appl. Crystallogr..

[64] Emsley P., Lohkamp B., Scott W.G., Cowtan K. (2010). Features and development of Coot. Acta Crystallogr. D Biol. Crystallogr..

[65] Murshudov G.N., Skubák P., Lebedev A.A., Pannu N.S., Steiner R.A., Nicholls R.A. (2011). REFMAC5 for the refinement of macromolecular crystal structures. Acta Crystallogr. D Biol. Crystallogr..

[66] Chen V.B., Arendall W.B., Headd J.J., Keedy D.A., Immormino R.M., Kapral G.J. (2010). MolProbity: all-atom structure validation for macromolecular crystallography. Acta Crystallogr. D Biol. Crystallogr..

[67] Chen S., Jiao L., Liu X., Yang X., Liu X. (2020). A dimeric structural scaffold for PRC2-PCL targeting to CpG Island chromatin. Mol. Cell.

