# Supplementary material for: CtBP1/2 oligomerization promotes G9a-Mediated transcriptional repression
Source: J Biol Chem. 2025 Dec 17;302(2):111063. doi: 10.1016/j.jbc.2025.111063 (PMC12809078; doi:10.1016/j.jbc.2025.111063)

Supplemental Data for

CtBP1/2 Oligomerization Promotes G9a-Mediated Transcriptional Repression

Bin Zhang[1,6], Junheng Jiang[1,6], Wenxin Sun[1,6], Shengwei Hu[1], Peihan Chen[1,2], Linsheng Li[1], Meizhi Jiang[1,4], Junjie Chen[1], Jinzhang Zeng[1], Dachuan Cai[3], Qiang Luo[3*], Wen Liu[1,5*], Qixu Cai[4*], Siming Chen[1,5*]

*Correspondence:

Siming Chen, simingchen@xmu.edu.cn

Qixu Cai, qxcai@xmu.edu.cn

Wen Liu, w2liu@xmu.edu.cn

Qiang Luo, qiangluo@hospital.cqmu.edu.cn

This PDF file includes:

Figures S1 to S5

Tables S1 to S3

KEY RESOURCES TABLE

REAGENT or RESOURCE	SOURCE	IDENTIFIER
**Antibodies**		
MYC tag Monoclonal antibody	Proteintech	Cat No. 60003-2-Ig
GAPDH Monoclonal antibody	Proteintech	Cat No. 60004-1-Ig
His Tag Monoclonal antibody	Proteintech	Cat No. 66005-1-Ig
HA tag Polyclonal antibody	Proteintech	Cat No. 51064-2-AP
FLAG tag Monoclonal Antibody	Sigma-Aldrich	Cat No. F1804
DiMethyl-Histone H3-K9 Rabbit mAb	Abclonal	Cat No. A26196
PTEN Rabbit mAb	Abclonal	Cat No. A19104
Phospho-Akt-S473 Rabbit mAb	Abclonal	Cat No. AP1208
CtBP2 Rabbit pAb	Abclonal	Cat No. A2257
EHMT2 Rabbit pAb	Abclonal	Cat No. A1247
Histone H3 Rabbit mAb	Abclonal	Cat No. A2348
Pan-Akt Rabbit mAb	Abclonal	Cat No. A18675
TriMethyl-Histone H3-K27 Rabbit mAb	Abclonal	Cat No. A22396
**Gel filter column**		
HiLoad™ 16/600 Superdex™ 200 pg	Cytiva	Cat No. 28989335
Capto™ Hires Q 5/50	Cytiva	Cat No. 29275878
Superose6™ Increase 10/300GL	Cytiva	Cat No. 29091596
**Recombinant DNA**		
pET-28a-SUMO-G9a (1-260)-FLAG	This Paper	N/A
pET-28a-SUMO-G9a (261-630)-FLAG	This Paper	N/A
pET-28a-SUMO-G9a (631-880)-FLAG	This Paper	N/A
pET-28a-SUMO-G9a (881-1210)-FLAG	This Paper	N/A
pET-28a-SUMO-G9a (881-913)-FLAG	This Paper	N/A
pET-28a-SUMO-G9a (913-1193)-FLAG	This Paper	N/A
pET-28a-SUMO-G9a (1193-1210)-FLAG	This Paper	N/A

pET-28a-SUMO-G9a (881-893)-FLAG	This Paper	N/A
pMAl-c2X-G9a (641-1193)$^{\Delta Pre\text{-}SET}$	This Paper	N/A
pMAl-c2X-G9a (641-1193)^{WT}	This Paper	N/A
pET-28a-SUMO-CtBP2 (1-445)	This Paper	N/A
pET-28a-SUMO-CtBP2 (1-445)$^{R147A/R148A/R169A/R177A}$	This Paper	N/A
pET-28a-SUMO-CtBP1 (1-440)	This Paper	N/A
pET-28a-SUMO-CtBP2 (31-364)	This Paper	N/A
pET-28a-SUMO-CtBP2 (31-364)-StrepI	This Paper	N/A
pET-28a-SUMO-G9a (881-913)^{A887R}-FLAG	This Paper	N/A
pET-28a-SUMO-G9a (881-913)^{D889R}-FLAG	This Paper	N/A
pET-28a-SUMO-G9a (881-913)^{T891R}-FLAG	This Paper	N/A
pET-28a-SUMO-G9a (881-913)^{P892E}-FLAG	This Paper	N/A
pET-28a-SUMO-GLP (1060-1125)-FLAG	This Paper	N/A
pET-28a-SUMO-GLP (350-500)-FLAG	This Paper	N/A
pLKO.1-shCtBP2 #1	This Paper	N/A
pLKO.1-shCtBP2 #2	This Paper	N/A
pLKO.1-shG9a #1	This Paper	N/A
pLKO.1-shG9a #2	This Paper	N/A
pLKO.1-shNC	This Paper	N/A
pCDH-EF1-3×Flag-G9a-(1-1210)	This Paper	N/A
pCDH-EF1-3×Flag-G9a-(1-1210)$^{\Delta Pre\text{-}SET}$	This Paper	N/A
pCDNA3.1-CtBP2-(1-445)-FLAG	This Paper	N/A
pCDNA3.1-G9a-(1-1210)$^{\Delta Pre\text{-}SET}$-Myc	This Paper	N/A
pCDNA3.1-G9a-(1-1210)-Myc	This Paper	N/A
pCDNA3.1-GLP-(1-1298)$^{\Delta Pre\text{-}SET}$-Myc	This Paper	N/A
pCDNA3.1-GLP-(1-1298)-Myc	This Paper	N/A
5×GAL4-UHRF1 Promoter-luciferase	Hai-Ning Du Laboratory	N/A

GAL4-HA	Hai-Ning Du Laboratory	
GAL4-HA-G9A^{wt}	Hai-Ning Du Laboratory	N/A
GAL4-HA-G9A$^{\Delta Pre\text{-}SET}$	This Paper	N/A
pCDNA3.0-Renilla	This Paper	N/A
psPAX2	Addgene	Cat No. 12260
pCMV-VSV-G	Addgene	Cat No. 8454
**REAGENT or RESOURCE**		
Polyethylenimine Linear (PEI) MW25000	YEASEN	Cat No. 40815ES03
Strep-Tactin®XT 4Flow® high capacity Resin	IBA Lifesciences	Cat No. 2-5030-010
FLAG® Peptide	Sigma	Cat No. F3290
Anti-DYKDDDDK G1 Affinity Resin	GenScript	Cat No. L00432-5
Puromycin (Solution 10 mg/mL)	YEASEN	Cat No.60209ES10
Hygromycin B	MCE	Cat No. HY-B0490
Clarity Western ECL Substrate, 500 mL	Bio-Rad	Cat No.1705061
Cell Counting Kit-8	YEASEN	Cat No.40203ES76
PrimeSTAR® GXL DNA Polymerase	TaKaRa	Cat No. R050A
Fetal bovine serum (FBS)	AMOBIO	Cat No. FBSAB001
Dulbecco's Modified Eagle Medium	Pricella	Cat No. PM150210
RPMI-1640	Pricella	Cat No. PM150110
**Deposited data**		
RNA-seq	This Paper	PRJNA1328908
Crystal structure of CtBP2-G9a	This Paper	PDB ID: 9WRI
**Bacterial Isolates**		
DH5α *E.coli* competent cells	Weidi	Cat. No. DL1004S
Rosetta2 (DE3) *E.coli* competent cells	Weidi	Cat. No. EC1014S
BL21 (DE3) competent cells	Weidi	Cat. No. EC1002S
**Experimental Models: Cell Lines**		

HCT116	ATCC	N/A
HEK-293T	ATCC	N/A
**Oligonucleotides**		
shG9a #1: GGACCTTCATCTGCGAGTATG	Sangon	N/A
shG9a #2: AGATTGAGCCTCCGCTGATTT	Sangon	N/A
shCtBP2 #1: CCTGAGAGTGATCGTGCGGAT	Sangon	N/A
shCtBP2 #2: CACTGCAATCTCAACGAACAT	Sangon	N/A
**Synthetic peptide**		
G9a (881-893) NKEGDTAWDLTPE	Hefei KS-V Peptide	N/A
G9a $(881\text{-}893)^{A887R}$ NKEGDTRWDLTPE	Hefei KS-V Peptide	N/A
G9a $(881\text{-}893)^{D889R}$ NKEGDTAWRLTPE	Hefei KS-V Peptide	N/A

RESOURCE AVAILABILITY

For further information and requests for resources or reagents, please contact the Lead Contact, Siming Chen (simingchen@xmu.edu.cn).

Table S1. Primers for RT-qPCR, Related to Method.

ID	Sequence	Source
PTEN-Fwd	5’-TGGATTCGACTTAGACTTGACCT-3’	Sangon
PTEN-Rev	5’-GGTGGGTTATGGTCTTCAAAAGG-3’	Sangon

Table S2. Primers for ChIP-qPCR, Related to Method.

ID	Sequence	Source
PTEN-Fwd	5’-GCAGGAAGGGTTGGGGTTCC-3’	Sangon
PTEN-Rev	5’-GGATACACGGGCCACAGTCG-3’	Sangon

Table S3. Crystallographic data collection and refinement statistics

**Data collection**	CtBP2/G9a
Space group	H32
Wavelength (Å)	0.97915
Unit cell parameters	
a, b, c (Å)	178.126, 178.126, 138.833
α, β, γ (°)	90, 90, 120
Resolution range (Å)	50-1.85 (1.88-1.85)
No. of unique reflections	71788 (3559)
Redundancy	17.1 (10.0)
I/σ	25.23 (1.07)
Completeness (%)	100.0 (100.0)
R_{merge}[a] (%)	15.7 (166.2)
$CC_{1/2}$	0.997 (0.551)
**Structure refinement**	
Resolution (Å)	1.85
R_{work}[b] (%)	16.70
R_{free}[c] (%)	20.16
RMSD bonds (Å)	0.008
RMSD angles (°)	1.597
Average B factor (Å^2)	30.0
No. of atoms	
Protein	5280
Ligand	88
Water	658
B factors (Å^2)	
Proteins	33.3
Ligand	24.3
Water	41.5
Ramachandran plot (%)	
Preferred	95.09
Allowed	4.61
Outliers	0.30

Numbers in parentheses represent the values for the highest-resolution shell.

[a]$R_{merge} = \Sigma|I_i - \langle I\rangle|/\Sigma I_i$, where I_i is the intensity of measured reflection and $\langle I\rangle$ is the mean intensity of all symmetry-related reflections.

[b]$R_{work} = \Sigma_W||F_{calc}| - |F_{obs}||/\Sigma|F_{obs}|$, where F_{obs} and F_{calc} are observed and calculated structure factors. W is working dataset of about 95% of the total unique reflections randomly chosen and used for refinement.

[c]$R_{free} = \Sigma_T||F_{calc}| - |F_{obs}||/\Sigma|F_{obs}|$, where T is a test dataset of about 5% of the total unique reflections randomly chosen and set aside prior to refinement.

Figure Legends:

Figure S1. Expression and purification of SUMO–CtBP2/1-FL.

(A) Gel-filtration profile of SUMO-CtBP2(1-445) on a HiLoad™ 16/600 Superdex™ 200 pg column equilibrated with 50 mM Tris-HCl (pH 8.0), 150 mM NaCl, and 2 mM DTT. The peak fractions containing the target protein were collected and analyzed by SDS-PAGE.

(B) SUMO-CtBP1(1-440) was purified using the same gel-filtration procedure, and the corresponding peak fractions were examined by SDS-PAGE.

(C) Secondary-structure prediction of the G9a Pre-SET domain using PSIPRED. Predicted α-helices are shown as pink bars; unstructured regions are indicated as random coil.

Figure S2. Sequence alignment of CtBP1 and CtBP2

Multiple sequence alignment was generated with CLUSTALW and visualized by ESPript 3.0. Residue conservation is highlighted following the default color scheme: darker shades indicate higher conservation, whereas lighter or uncolored residues denote divergence. The extensive color coverage across both sequences underscores the strong evolutionary conservation between CtBP1 and CtBP2.

Figure S3. G9a and GLP engage CtBP2 through distinct domains

(A) Multiple sequence alignment (CLUSTALW) of the Glu-Cys domains from human G9a and GLP, rendered with ESPript 3.0. Conservation is color-coded by identity (darker = higher). A DLS-like CtBP-binding motif, present in GLP but absent in G9a, is indicated with a blue dashed box and blue asterisks, suggesting that this region of G9a does not contribute to CtBP1/2 binding.

(B) Alignment of the Pre-SET domains of G9a and GLP using the same color scheme as in (A). The Pre-SET segments are poorly conserved (blue dashed box and asterisks), indicating that this region of GLP does not participate in CtBP1/2 recognition.

(C) In vitro FLAG pull-down assay using the Pre-SET domains of G9a and GLP.

Recombinant SUMO-G9a (881–913)-FLAG or SUMO-GLP (1060–1125)-FLAG was incubated with SUMO-CtBP2(1–445). Bound proteins were captured with anti-FLAG resin and analyzed by SDS-PAGE. The G9a Pre-SET fragment, but not the GLP counterpart, exhibited detectable binding to CtBP2.

(D) Pull-down with SUMO-GLP (300–500)-FLAG (encompassing the Glu-Cys domain) efficiently retrieved SUMO-CtBP2(1–445), demonstrating that the GLP Glu-Cys domain is necessary and sufficient for CtBP2 interaction.

Figure S4. Purification of CtBP2(31-364) and CtBP1(28-378) for crystallization

(A) Anion-exchange purification of human CtBP2(31-364) on a Capto™ HiRes Q 5/50 GL column (Cytiva). Low-salt buffer: 50 mM Tris-HCl pH 8.0, 100 mM NaCl, 2 mM DTT; high-salt buffer: 50 mM Tris-HCl pH 8.0, 1 M NaCl, 2 mM DTT. A linear NaCl gradient was applied, and fractions corresponding to the major peak (monitored at 280 nm, blue) were pooled and analyzed by SDS-PAGE (inset).

(B) The pooled CtBP2(31-364) fractions were concentrated to 1 mL and subjected to size-exclusion chromatography on a Superose™ 6 Increase 10/300 GL column in 50 mM Tris-HCl pH 8.0, 100 mM NaCl, 2 mM DTT. Peak fractions were analyzed by SDS-PAGE (inset).

(C) Relative luciferase activity in HEK293T cells transfected with GAL4-HA-G9a(WT) together with either oligomeric CtBP2 or monomeric CtBP2, normalized to vector controls. Bar graphs represent the mean ± SD of three independent biological replicates. Statistical significance was determined using unpaired t-test or one-way ANOVA as appropriate. n.s., not significant; $p < 0.05$ (*); $p < 0.01$ (**) ($n = 3$ biological replicates).

(D) Immunoblot analysis verifying comparable expression levels of GAL4-HA-G9a(WT) and wild-type or mutant CtBP2 constructs used in panel (C). GAPDH serves as the loading control.

Figure S5. Overlap between G9a knockdown and rescue transcriptomes

(A) A Venn diagram showing the overlap between genes altered by G9a knockdown and those restored upon re-expression of wild-type G9a in G9a-depleted HCT116 cells.

(B) Western blot analysis of CtBP2-knockdown HCT116 cells reconstituted with shRNA-resistant wild-type ($CtBP2^{Oligomer}$) or monomeric ($CtBP2^{Monomer}$) constructs. Both were expressed at levels comparable to endogenous CtBP2 in shNC controls (GAPDH as loading control).

(C-D) Colony formation assays and quantification showing that $CtBP2^{Oligomer}$, but not $CtBP2^{Monomer}$, rescued the proliferation defect caused by CtBP2 depletion. Bar graphs represent the mean ± SD of three independent experiments. Statistical significance was determined using unpaired t-test or one-way ANOVA as appropriate. n.s., not significant; $p < 0.05$ (*); $p < 0.01$ (**) (n = 3 biological replicates).

Figure S6. Rescue experiments in CtBP2-knockdown HCT116 cells

(A-B) Rescue experiments in CtBP2-knockdown HCT116 cells. Re-expression of CtBP2(WT), but not the monomeric CtBP2 mutant, restored PTEN repression, reactivated AKT phosphorylation, and recovered H3K9me2 enrichment at the PTEN promoter. These results demonstrate that CtBP2 oligomerization is required to cooperate with G9a in promoting PTEN repression and H3K9me2 deposition. Bar heights represent mean values from three independent experiments, and error bars denote the standard deviation (SD). Statistical analyses were performed using unpaired t-tests or one-way ANOVA. as appropriate. n.s., not significant; $p < 0.05$ (*); $p < 0.01$ (**), (n = 3 biological replicates).

(C-D) Quantitative analysis of PTEN expression (C) and AKT phosphorylation (D) from Fig. 6G. Band intensities from Western blots were determined using ImageJ, normalized to the shNC group, and expressed as ratios relative to GAPDH or total AKT, respectively. Data represent mean ± SD (n=3). Significance was assessed by unpaired t-test (*$p < 0.05$, **$p < 0.01$; n.s., not significant).

Fig.S1 (Zhang et al)

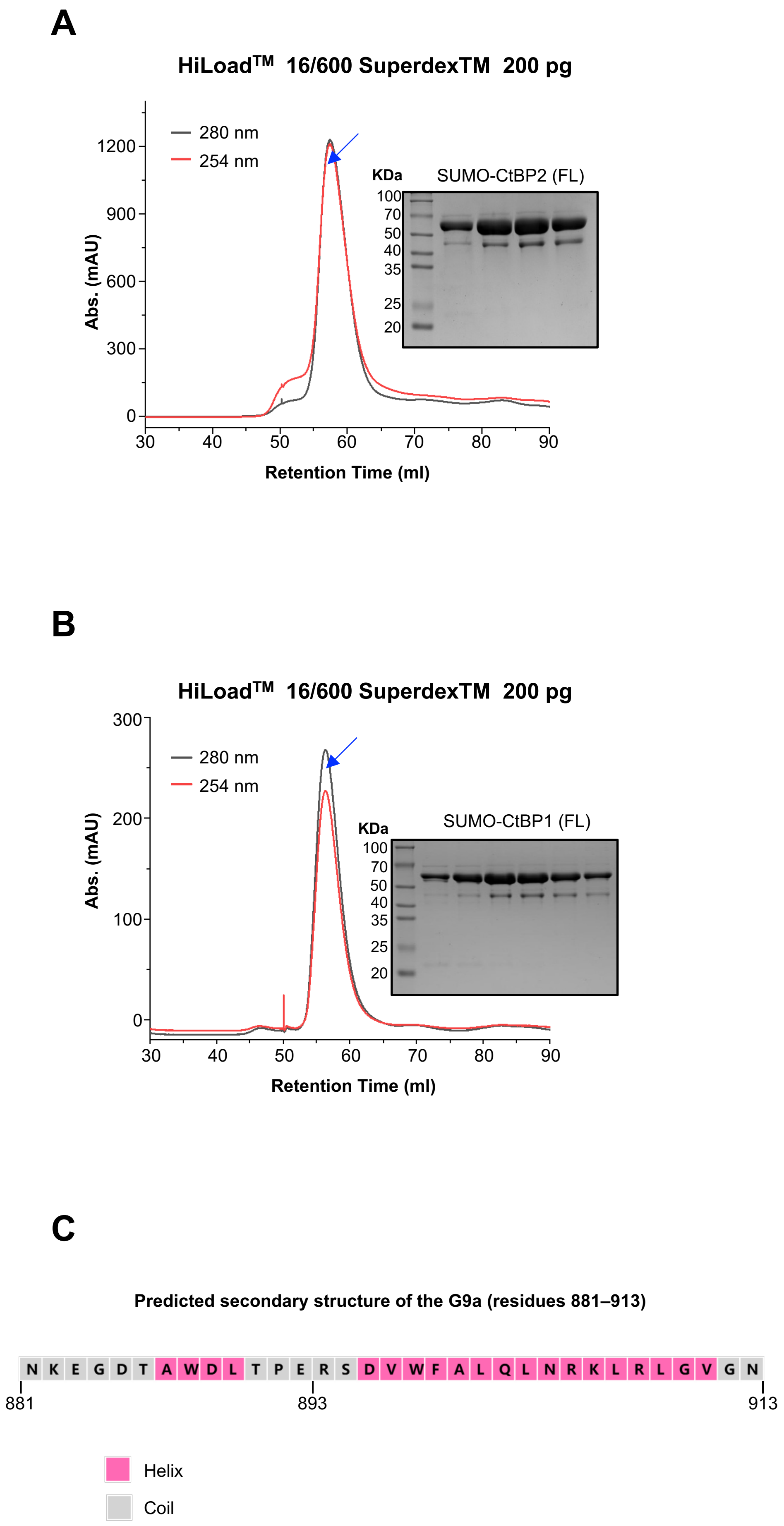

Fig.S2 (Zhang et al)

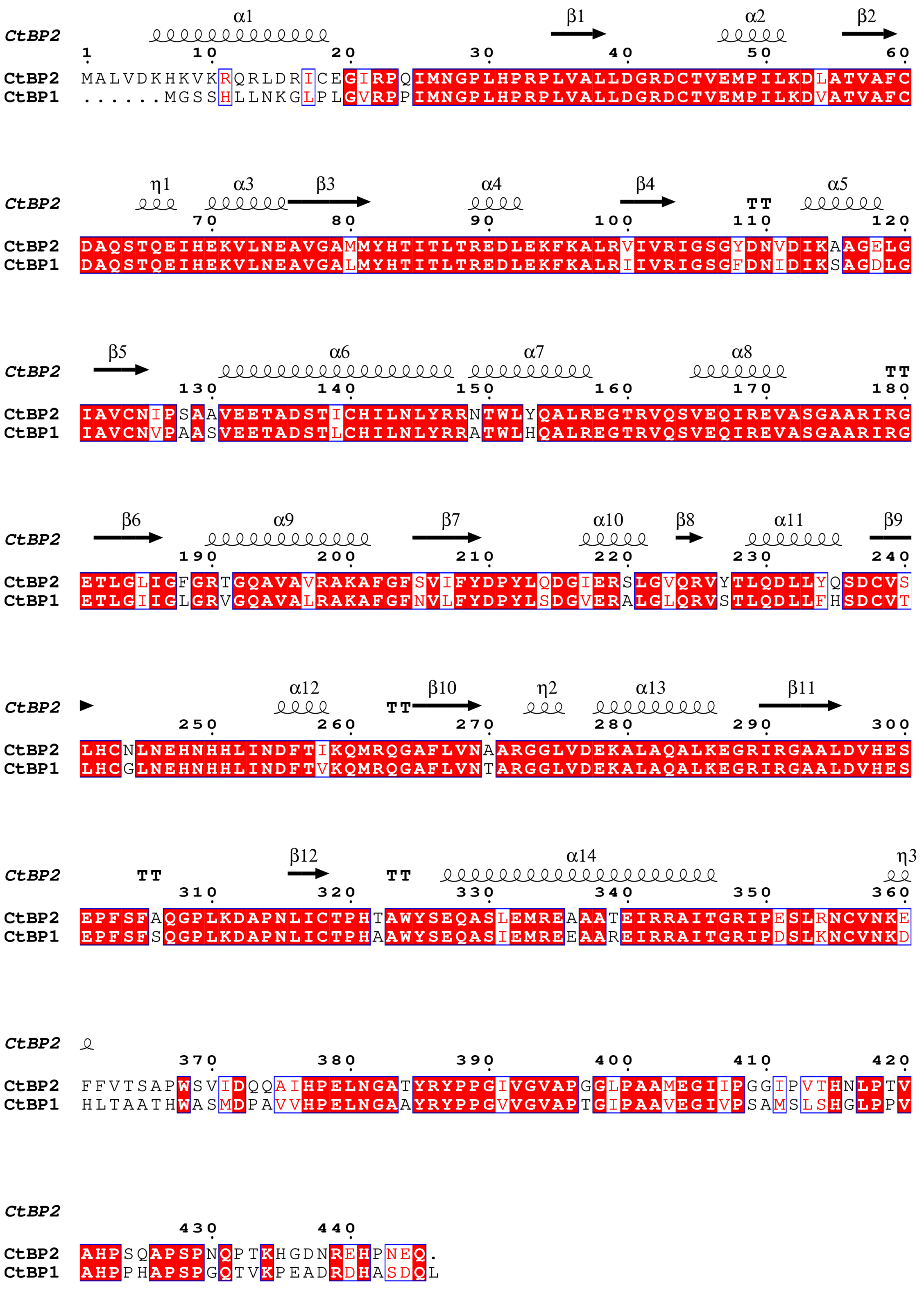

Fig.S3 (Zhang et al)

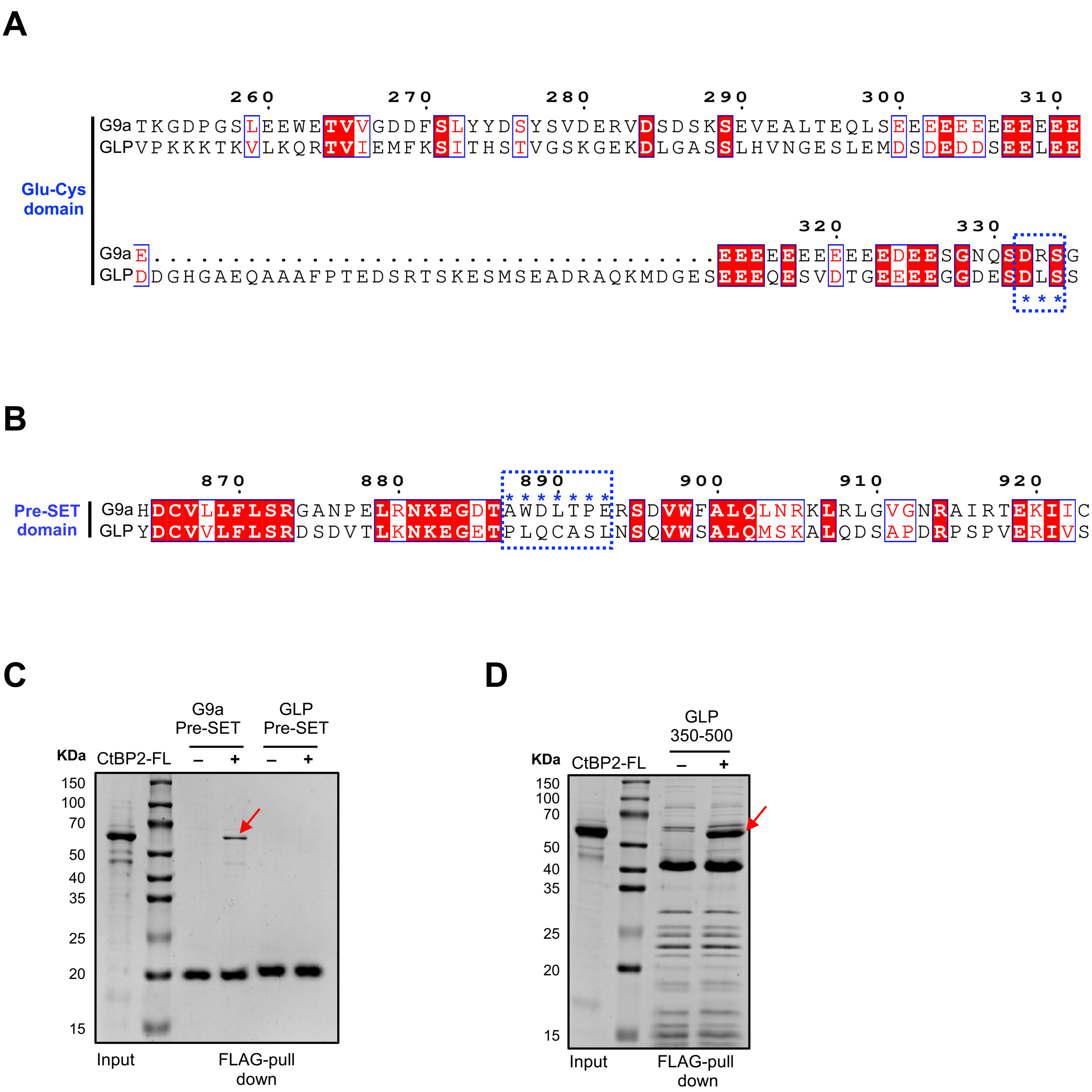

Fig.S4 (Zhang et al)

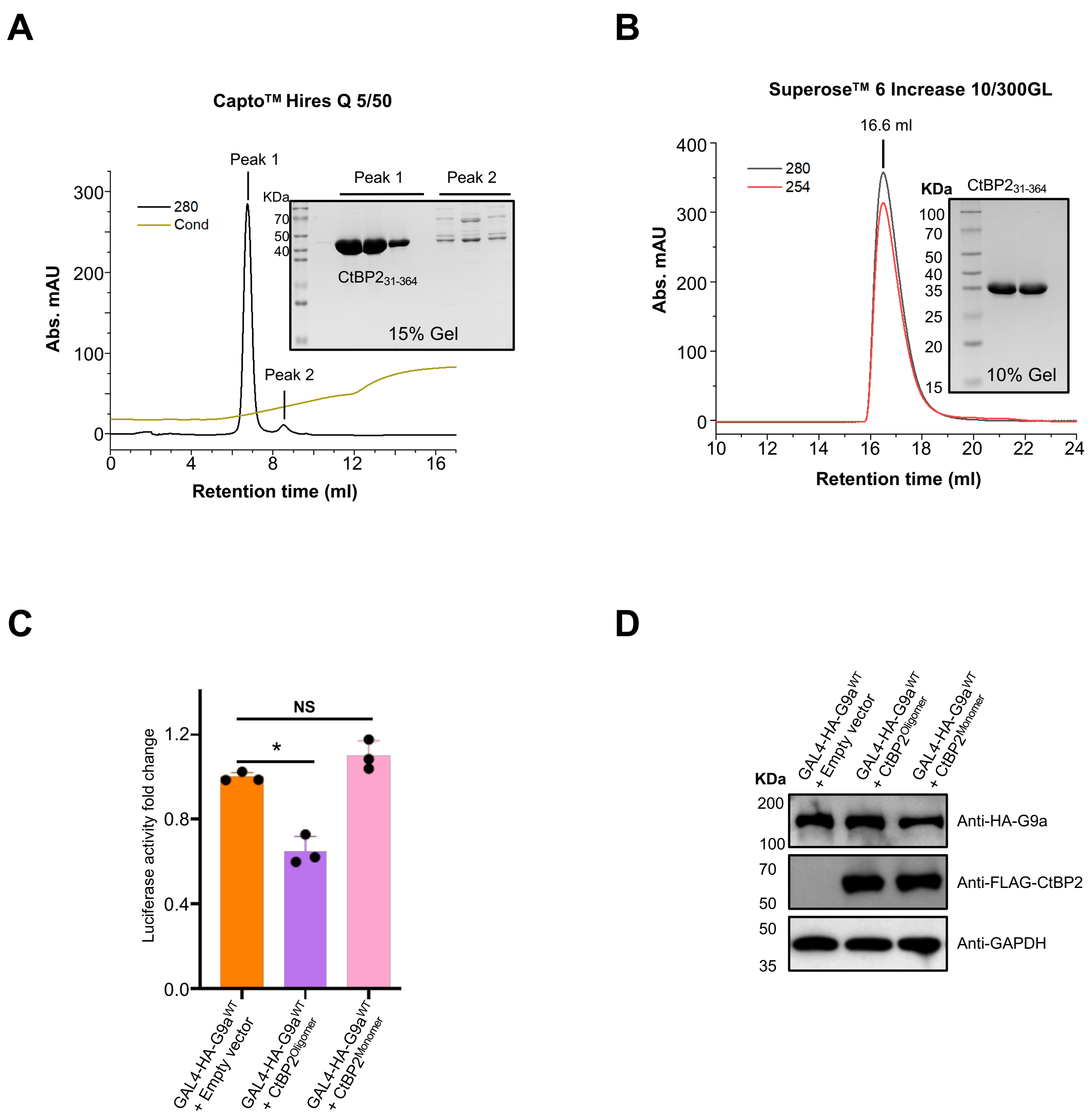

Fig.S5 (Zhang et al)

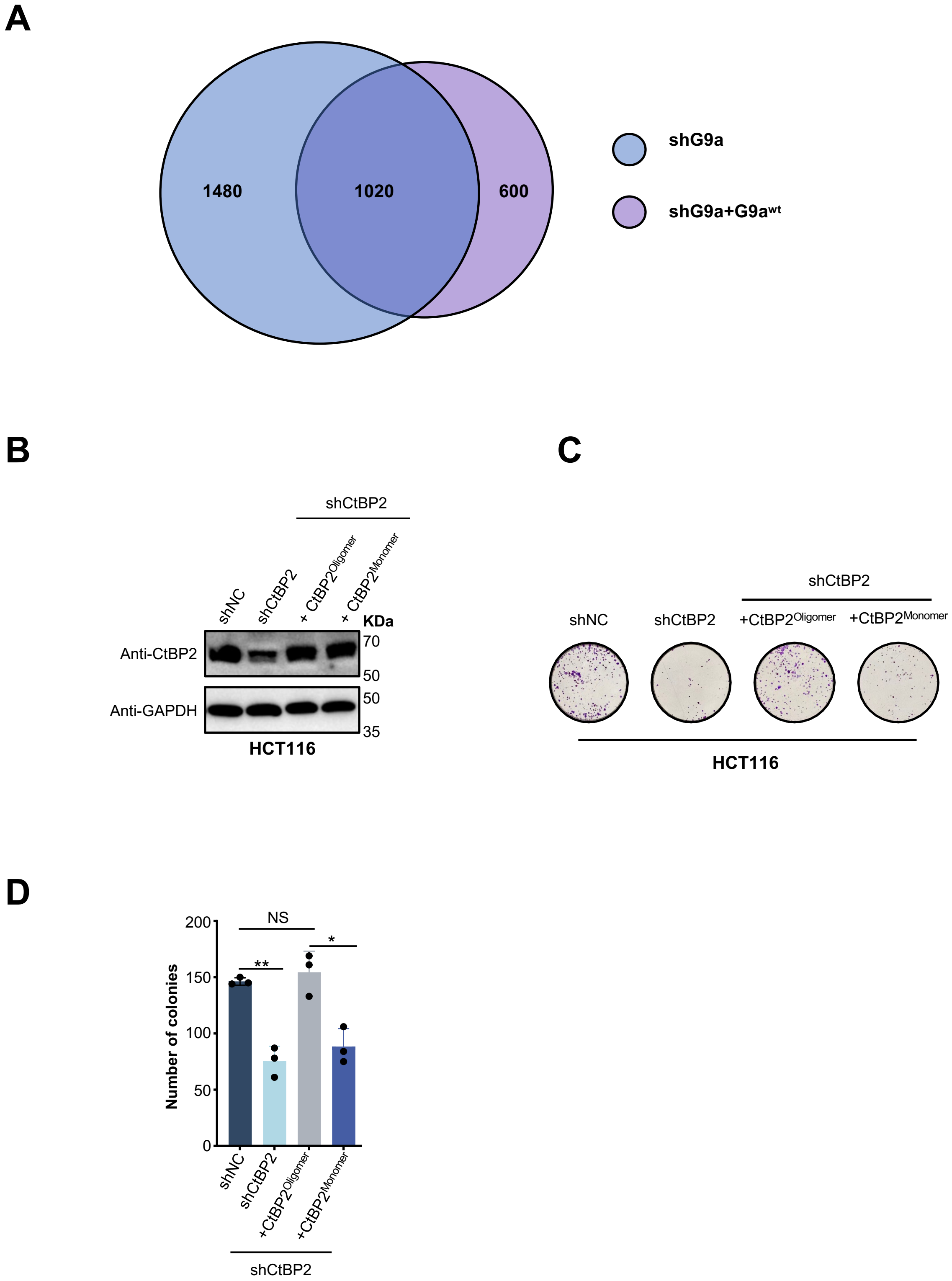

Fig.S6 (Zhang et al)

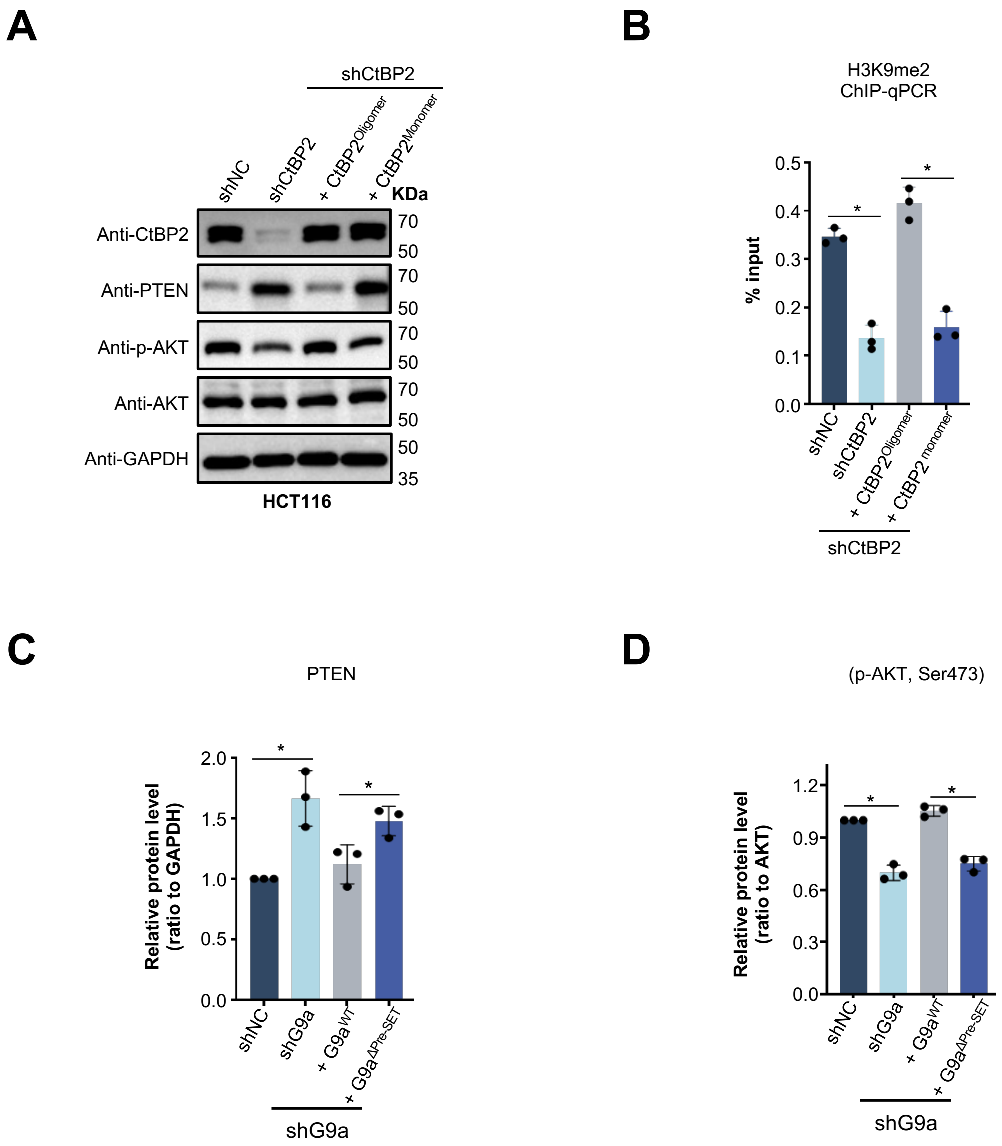

Supplement: Supporting information [file mmc1.pdf]
